# Hedgehog signaling is a promising target for the treatment of hepatic fibrogenesis: a new management strategy using itraconazole-loaded nanoparticles

**DOI:** 10.3389/fphar.2024.1377980

**Published:** 2024-05-14

**Authors:** Elsayed A. Elmorsy, Sameh Saber, Ahmed Y. Kira, Abdullah Alghasham, Mohamed R. Abdel-Hamed, Maha M. Amer, Enas A. Mohamed, A AlSalloom A., Abdullah S. Alkhamiss, Rabab S. Hamad, Mustafa Ahmed Abdel-Reheim, Abousree T. Ellethy, Hossam A. Elsisi, Mansour Alsharidah, Sahar R. Elghandour, Tayseer Elnawawy, Rasha Abdelhady

**Affiliations:** ^1^ Department of Pharmacology and Therapeutics, College of Medicine, Qassim University, Buraydah, Saudi Arabia; ^2^ Department of Clinical Pharmacology, Faculty of Medicine, Mansoura University, Mansoura, Egypt; ^3^ Department of Pharmacology, Faculty of Pharmacy, Delta University for Science and Technology, Gamasa, Egypt; ^4^ Department of Pharmaceutics, Faculty of Pharmacy, Delta University for Science and Technology, Gamasa, Egypt; ^5^ Department of Anatomy, College of Medicine, Qassim University, Buraydah, Saudi Arabia; ^6^ Department of Anatomy and Embryology, Faculty of Medicine, Ain Shams University, Cairo, Egypt; ^7^ Department of Anatomy, Faculty of Medicine, Cairo University, Cairo, Egypt; ^8^ Department of Pathology, College of Medicine, Qassim University, Buraydah, Saudi Arabia; ^9^ Biological Sciences Department, College of Science, King Faisal University, Saudi Arabia; ^10^ Central Laboratory, Theodor Bilharz Research Institute, Giza, Egypt; ^11^ Department of Pharmaceutical Sciences, College of Pharmacy, Shaqra University, Shaqra, Saudi Arabia; ^12^ Department of Pharmacology and Toxicology, Faculty of Pharmacy, Beni-Suef University, Beni Suef, Egypt; ^13^ Department of Oral and Medical Basic Sciences, Biochemistry Division, College of Dentistry, Qassim University, Buraydah, Saudi Arabia; ^14^ Department of Pharmacology and Toxicology, College of Pharmacy, Qassim University, Buraydah, Saudi Arabia; ^15^ Department of Clinical Pharmacology, Faculty of Medicine, Zagazig University, Zagazig, Egypt; ^16^ Department of Physiology, College of Medicine, Qassim University, Buraydah, Saudi Arabia; ^17^ Department of Anatomy and Histology, College of Medicine, Qassim University, Buraydah, Saudi Arabia; ^18^ Department of Pharmaceutics, Egyptian Drug Authority, Cairo, Egypt; ^19^ Pharmacology and Toxicology Department, Faculty of Pharmacy, Fayoum University, Fayoum, Egypt

**Keywords:** liver fibrosis, itraconazole, hedgehog signaling, anti-fibrotic, inflammation, nanotechnolody

## Abstract

Liver fibrosis is a disease with a great global health and economic burden. Existing data highlights itraconazole (ITRCZ) as a potentially effective anti-fibrotic therapy. However, ITRCZ effect is hindered by several limitations, such as poor solubility and bioavailability. This study aimed to formulate and optimize chitosan nanoparticles (Cht NPs) loaded with ITRCZ as a new strategy for managing liver fibrosis. ITRCZ-Cht NPs were optimized utilizing a developed 2^2^ full factorial design. The optimized formula (F3) underwent comprehensive *in vitro* and *in vivo* characterization. *In vitro* assessments revealed that F3 exhibited an entrapment efficiency of 89.65% ± 0.57%, a 169.6 ± 1.77 nm particle size, and a zeta potential of +15.93 ± 0.21 mV. Furthermore, *in vitro* release studies indicated that the release of ITRCZ from F3 adhered closely to the first-order model, demonstrating a significant enhancement (*p-value* < 0.05) in cumulative release compared to plain ITRCZ suspension. This formula increased primary hepatocyte survival and decreased LDH activity *in vitro*. The *in vivo* evaluation of F3 in a rat model of liver fibrosis revealed improved liver function and structure. ITRCZ-Cht NPs displayed potent antifibrotic effects as revealed by the downregulation of TGF-β, PDGF-BB, and TIMP-1 as well as decreased hydroxyproline content and α-SMA immunoexpression. Anti-inflammatory potential was evident by reduced TNF-α and p65 nuclear translocation. These effects were likely ascribed to the modulation of Hedgehog components SMO, GLI1, and GLI2. These findings theorize ITRCZ-Cht NPs as a promising formulation for treating liver fibrosis. However, further investigations are deemed necessary.

## 1 Introduction

Hepatic fibrosis is a catastrophic consequence of iterative hepatic tissue damage caused by several etiologies (e.g., viral hepatitis, autoimmune and alcoholic liver disease) that eventually leads to compromised liver function. Liver fibrosis represents a principal worldwide health and economic burden accounting for significant global mortality and morbidity (Roehlen et al., 2020). Potential clinical complications of liver fibrosis include progression to liver cirrhosis (ranked the 10th most common cause of death in Africa) (Devarbhavi et al., 2023), portal hypertension, hepatic encephalopathy, or in the worst case hepatocellular carcinoma (D'Amico et al., 2018; Asrani et al., 2019). Other than liver transplantation, there is no proven cure for end-stage liver fibrosis. Therefore, the development of novel antifibrotic therapeutic interventions, targeting dysregulated signaling cascades, implicated in hepatic fibrogenesis is a crucial unmet need.

The main pathological hallmarks of liver fibrosis include the destruction of the original hepatic lobular structure and formation of fibrous scarring mainly consequent to excessive production and accumulation of liver degrading extracellular matrix (ECM) (Iredale, 2007; Xu et al., 2016). Significantly, ECM produced during liver fibrosis displayed type I collagen- and fibronectin I-rich composition and chiefly secreted by activated hepatic stellate cells (HSCs), the main effector cells in hepatic fibrogenesis. Notably, injured hepatic tissues stimulate quiescent HSCs differentiation into collagen-producing myofibroblast-like phenotype alongside the activation of resident fibroblasts trans-differentiation into α-smooth muscle actin (α-SMA) expressing myofibroblasts (Elpek, 2014; Zhou et al., 2014).

On the molecular basis, aberrant dysregulations of morphogenic signaling cascades were reported to orchestrate liver fibrogenesis, among those, Hedgehog was stated to be crucial to the development and progression of fibrogenic events in the liver. Of note, Hedgehog is a chief fibrogenic pathway that modulates HSCs fate (epithelial-to-mesenchymal transitions and *vice versa*) (Xie et al., 2013). It encompasses various protein components including the signal transducer smoothened (SMO) and glioma-associated oncogene homolog (GLI) transcriptional factors (GLI1 and 2). Mechanistically, in normal hepatic tissues, the Hedgehog pathway is not activated (Chuang and McMahon, 1999). Meanwhile, the activation takes place in response to hepatic damage where excessive Hedgehog pathway activation contributes significantly to the hepatic fibrotic microenvironment through triggering HSCs activation (Du et al., 2018; Wu et al., 2021). Remarkably, the Hedgehog signaling pathway orchestrates a complex network of morphogen signaling trajectories, including transforming growth factor beta (TGF-β), controlling cell fate acquisition and hepatic fibrotic mechanisms (Omenetti et al., 2011). Thus, the modulation of Hedgehog trajectory could dampen the activity of other cascades enabling attenuation of hepatic fibrosis.

Itraconazole (ITRCZ), is a broad-spectrum triazole antifungal widely used in clinical settings. Since ITRCZ is a well-tolerated drug, this recommends its repositioning for other off-label uses. Notably, ITRCZ has demonstrated remarkable antifibrotic activity in a previous *in-vitro* study that reported the capacity of ITRCZ to suppress myofibroblast activation (Bollong et al., 2017a). Additionally, previous data highlighted ITRCZ as a potential anti-fibrotic therapy for a range of fibrotic diseases including liver fibrosis (Bollong et al., 2017b). Nevertheless, ITRCZ has many pharmaceutical drawbacks. As a BCS class II medication, it demonstrates low solubility and limited bioavailability (Buchanan et al., 2007). Previous investigations have discovered various formulation strategies to overcome the limitations associated with ITRCZ use, including solid dispersion (Engers et al., 2010), emulsification (Hong et al., 2006), complexation (Buchanan et al., 2007), and different nanoformulations (Buescher et al., 2023; Wang et al., 2023; Yousaf et al., 2023).

We have developed and optimized chitosan nanoparticles loaded with itraconazole (ITRCZ-Cht NPs) in the current research. This formulation serves to overcome the limitations of the drug and augment the effectiveness of ITRCZ for managing liver fibrosis. The selection of chitosan as the delivery system is based on its distinctive properties of biodegradability, biocompatibility, and mucoadhesive characteristics. These properties facilitate prolonged residence time and enhanced drug permeation (Fathima et al., 2022; Manna et al., 2023). Moreover, Cht NPs offer the benefits of liver targeting due to the unique structural features of liver tissue. Abundant blood supply and phagocytic cells make the liver a primary organ for NP accumulation (Jenne and Kubes, 2013). The size and cationic surface charge of Cht NPs also enable the passive targeting into liver tissues (Bonferoni et al., 2020; Fathima et al., 2022).

Numerous studies investigating Cht NPs have granted promising conclusions in increasing drug accumulation within the liver highlighting their potential within diverse therapeutic applications for hepatic disorders (Loutfy et al., 2020; Nasr et al., 2023b; Moustafa et al., 2023; Sahyon et al., 2023). This research presents the first exploration of repurposing ITRCZ for the management of liver fibrosis as well as the first to report the utilization of ITRCZ-Cht NPs in this condition. Given this aim, ITRCZ-Cht NPs were prepared and optimized utilizing a 2^2^ full factorial design. This optimized formulation underwent *in vitro* and *in vivo* characterization. *In vitro* assessments included the determination of thermal and morphological properties as well as the evaluation of physical stability and *in vitro* release. Our *in vivo* evaluation was performed to ascertain the efficacy of ITRCZ-Cht NPs in mitigating liver fibrosis in a rat model induced by thioacetamide (TAcA) emphasizing the role of Hedgehog signaling. Anticipations were guided towards the potential enhancement of ITRCZ efficacy compared with plain ITRCZ.

## 2 Methods

### 2.1 Design and preparation of ITRCZ-Cht NPs

#### 2.1.1 Full factorial design

We employed a full factorial design to optimize the preparation of ITRCZ (Sigma)-Cht NPs, specifically a two-factor two-level (2^2^) configuration. The dependent variables selected for optimization were entrapment efficiency (EE%), zeta potential (ZP), and particle size (PS), as outlined in Table 1. Based on thorough preliminary studies, Cht concentration (A) and Na-TPP concentration (B) were designated as the independent variables, each with two levels (0.2% and 0.3% w/v) and (1% and 1.5% w/v), respectively. All possible combinations of these factors were systematically tested, resulting in a total of four formulations, as detailed in Table 2.

**TABLE 1:** The encoded units representing levels and their corresponding variables in the 2^2^ full factorial design.

Independent variables	Levels	
	Low	High
A = Cht concentration (% w/v)	0.2	0.3
B = Na-TPP concentration (% w/v)	1	1.5
Coded values	−1	+1
Dependent variables (responses)	Target	
R1 = EE%	Maximize	
R2 = ZP	Maximize	
R3 = PS	Minimize	

**TABLE 2:** The matrix design for the formulated ITRCZ-CH NPs.

Formulation code	Coded levels		Cht concentration	Na-TPP concentration
	A	B		
F1	−1	−1	0.2% w/v	1% w/v
F2	+1	−1	0.3% w/v	1% w/v
F3	−1	+1	0.2% w/v	1.5% w/v
F4	+1	+1	0.3% w/v	1.5% w/v

#### 2.1.2 Preparation of ITRCZ-Cht NPs

Based on the aforementioned design, four distinct formulations were developed using the ionic gelation method (Calvo et al., 1997). Cht solutions (20 mL) were prepared by dissolving Cht in 10% v/v acetic acid (pH = 2.5 ± 0.1). Afterward, an aliquot of 1 mL of ITRCZ solution in DMSO equivalent to 20 mg of ITRCZ was introduced to the Cht solutions, and the mixture was stirred continuously (600 rpm) for 15 min at room temperature (RT). Na-TPP solutions (8 mL) were prepared by dissolving Na-TPP in deionized water and dropping wisely to the drug-chitosan mixture with constant stirring (600 rpm) at RT. The formation of NPs arose instantly upon the addition of the Na-TPP solution. The nanodispersion was stirred for a further 1 hour to facilitate cross-linking. Subsequently, the NPs underwent 10 min of sonication in a bath sonicator before analysis. ITRCZ-free NPs (plain NPs) were produced under identical conditions for control purposes.

### 2.2 Characterization of ITRCZ-Cht NPs

#### 2.2.1 Measurement of the entrapment efficiency of ITRCZ-Cht NPs

To assess the EE% of ITRCZ-Cht NPs, 0.5 mL of the formulated nanodispersions were combined with glacial acetic acid (1 mL) to dissolve the polymeric coating, facilitating the determination of the total drug amount (*A*_*1*_). The amount of non-entrapped ITRCZ (*A*_*2*_) was determined by subjecting 0.5 mL of the formulations to cooling centrifugation for 1 h at 15,000 rpm, followed by the isolation of the supernatant. The EE% of the ITRCZ-Cht NPs were calculated using the following equation: $EE\;\left(\%\right)=\frac{A_{1}-\;A_{2}}{A_{1}}\times 100$ . The quantification of the ITRCZ amount was conducted using an RF-6000 spectrofluorometer (Shimadzu, Japan) (excitation (λ_ex_) at 260 nm and emission (λ_em_) at 380 nm) (Elmansi et al., 2020). A linear calibration curve covering the concentration range of 0.2–200 μg/mL was established, yielding a high correlation coefficient (*R* ^2^) of 0.9997. To eliminate potential interference from NPs’ components during determinations. Plain NPs were utilized as blank.

#### 2.2.2 Measurement of polydispersity index, particle size, and zeta potential

We utilized the method of electrophoretic light scattering to determine the ZP value, while the dynamic light scattering method was used to determine the PS and PDI of the prepared formulations utilizing a Zetasizer (Malvern, UK). Prior to each measurement, deionized water was used to dilute the samples at a 1:20 ratio.

### 2.3 Optimization of the prepared ITRCZ-Cht NPs

The response optimization technique was used after the factorial experimental design with Minitab 21 software to identify the optimal conditions for choosing the utmost optimized formulation. The optimization process’s objective was completed by achieving the minimum PS while simultaneously maximizing the ZP and EE values (Table 2). Consequently, the selected formulation underwent centrifugation, and the NPs were washed after being collected with deionized water and subjected to freeze-drying for further characterization.

### 2.4 Characterization of the optimized formula

Characterization of the optimized formula was done through a methodical analysis of various parameters. The loading efficiency (LD%) was determined using the same methodology for measuring EE%. However, LD% was calculated by assessing the weight of entrapped ITRCZ relative to the total weight of the NPs. We employed a transmission electron microscope (TEM) (model 2,100, JEOL, Japan) to elucidate the morphological attributes, such as shape and size. The thermal characteristics of both ITRCZ and the lyophilized optimized formulation were investigated using differential scanning calorimetry (DSC) (DSC-60, Shimadzu, Japan). Approximately 5 mg of each sample was introduced into a nitrogen atmosphere, and the analysis was conducted with a heating rate of 10°C/min over a temperature range from 0°C to 300°C. The investigation into the *in vitro* release profiles of ITRCZ from the optimized formulation and plain suspension was conducted using a release medium comprising PBS with a pH of 7.4, supplemented with sodium lauryl sulfate (0.5% w/v) to enhance the solubility of ITRCZ, following established methodologies (Alhakamy and Md, 2019; Alhowyan et al., 2019). Each of the TRCZ-Cht NPs and plain ITRCZ suspension, equivalent to 1 mg ITRCZ, was introduced into dialysis bags (molecular weight cutoff = 12 kDa) and immersed in 200 mL of the release medium. The system was retained at 37°C while stirring at 100 rpm for 24 h. At specified intervals over the 24 h, 2 mL samples were withdrawn and replaced with an equal volume of fresh buffer solution. The released ITRCZ was quantified spectrofluorimetrically at λem of 380 nm after excitation at 260 nm. Subsequently, the obtained ITRCZ release data were subjected to kinetic analysis using DDsolver, a software integrated with Microsoft Excel. The release kinetics of the optimized formulation were evaluated using zero-order, first-order, and Higuchi models. The optimal release kinetics model was determined based on the model exhibiting the highest *R* ^*2*^ value. The impact of storage conditions on the physical attributes of the optimized lyophilized formula was investigated by packaging the formulation into vials and subjecting it to distinct storage environments for a duration of 3 months, encompassing room temperature conditions (21°C–25°C) and refrigerated conditions (2°C–6°C). Subsequently, various parameters, including PS, PDI, ZP, and EE%, were assessed monthly. Before each determination, the lyophilized samples were reconstituted using PBS (pH = 7.4).

### 2.5 Liver partition coefficient (Kp, liver) determination

The liver targeting efficacy of ITRCZ-Cht NPs was assessed by determining the liver partition coefficient (*Kp, liver*). This coefficient was calculated by calculating the ratio of ITRCZ concentration in the liver (*C*_*liver*_) to that in the plasma (*C*_*plasma*_) at four definite time intervals (0.5, 2, 4, and 8 h). Twenty-four male adult Sprague Dawley rats (220–240 g) were randomly allocated to two groups, each comprising 12 rats. All rats were maintained in the same environmental conditions and received the same rodent chow diet. Intraperitoneal administration of plain ITRCZ and ITRCZ-Cht NPs dispersed in PBS pH 7.4 was done. The ITRCZ dose was set at 10 mg/kg. Blood samples were collected and processed to obtain plasma. Subsequently, rats’ livers were separated after euthanasia, weighed, and homogenized using an Ultra Turrax homogenizer (IKA, Werke GmbH and Co. KG, Germany) with the addition of 10 mL of methanol. To determine the concentration of ITRCZ in plasma and liver homogenates, 0.5 mL of the respective homogenates or plasma were spiked with 0.5 mL of different ITRCZ working solutions (0.5–5 μg/mL) and 4 mL of methanol. Following a thorough deproteinization period and vortex mixing, the liquid was centrifuged for 15 min at 3,000 rpm. After passing through a 0.22 μm filter, a 1 mL aliquot of the supernatant was placed into a calibrated 10 mL volumetric flask. To attain final ITRCZ concentrations ranging from 50–500 ng/mL, deionized water was then added. After illumination at 260 nm, the relative fluorescence intensity of the solutions was measured at λem of 380 nm. To get rid of matrix effects, liver homogenate samples and blank plasma were processed in the same way. Both the liver homogenate and plasma calibration curves had an R2 of 0.9994, indicating that they were linear. The method’s great accuracy was confirmed by the validation findings, which showed a mean recovery percentage of 99.06 ± 2.25 percent for liver homogenate and 98.79 ± 1.03 percent for plasma. The reproducibility of the procedure was demonstrated by the intra- and inter-day precision, with RSD percent values less than 2 percent.

### 2.6 Isolation of primary rat hepatocytes

A male adult Sprague Dawley rat (220 g) purchased from Theodor Bilharz Research Institute was used to perform a two-step collagenase perfusion method. The resultant cells were suspended in DMEM and then centrifuged at 50 *g* for 3 min at 4°C. After three cycles of careful washing, the cells were resuspended in 25 mL DMEM and 25 mL Percoll solution in PBS. After centrifugation for 10 min at 200 × *g* and 4°C, the live cells settled up at the bottom and were consequently resuspended in 20 mL of DMEM at 37°C. Finally, the viability was evaluated by the Trypan blue dye exclusion method.

### 2.7 Assessment of the effect of ITRCZ-Cht NPs on the viability of primary rat hepatocytes

Using DMEM as the culture medium, primary rat hepatocytes were cultured in 96-well culture plates at a density of 2 × 10^4^ cells per well. After a 24-h culturing period, the medium was replaced with a new one containing various concentrations of ITRCZ. To determine the cytotoxicity, an MTT assay was conducted, and the optical density of each well was determined at 595 nm. Each concentration was investigated in triplicate and the concentration that preserved viability at about 85% following 24 h incubation was considered safe for assessment of the hepatocyte survival. In this context, 10 µM ITRCZ was selected to investigate the capacity of ITRCZ-Cht NPs to mitigate TAcA noxious efects compared to plain ITRCZ. Primary rat hepatocytes were seeded in 96-well plates at a density of 2 × 10^4^. In CTR group, primary rat hepatocytes were treated with DMEM for 24 h; the ITRCZ-Cht NPs group was treated with the DMEM and ITRCZ-Cht NPs (10 µM); The TAcA group was treated with DMEM comprising TAcA (100 mM) (Sigma-Aldrich); the TAcA/ITRCZ group was treated with DMEM comprising TAcA (100 mM) and plain ITRCZ (10 µM); TAcA/ITRCZ-Cht NPs group was treated with DMEM comprising TAcA (100 mM) and ITRCZ-Cht NPs (10 µM). Each group was tested in triplicate followed by an MTT assay to determine the cell viability (percentage).

### 2.8 Assessment of lactate dehydrogenase (LDH) in primary rat hepatocytes

A parallel set of groups was conducted for LDH activity assay as instructed by Sigma-Aldrich.

### 2.9 Animal study

Male adult Sprague Dawley rats (220–240 g) were purchased from Theodor Bilharz Research Institute and used for the study following the guidelines from the research ethics committee at Delta University (approval number: 16/2022,4). The rats were assigned into five groups as that manner: CTR (*n* = 6), serve as normal rats; ITRCZ-Cht NPs (*n* = 6), rats received ITRCZ-Cht NPs (10 mg/kg/day i. p.); TAcA (*n* = 10), rats received biweekly TAcA (Sigma) at 150 mg/kg i. p. For a total of 6 weeks (Yang et al., 2019; Mohammed, 2023); TAcA/ITRCZ (*n* = 10), rats received biweekly TAcA and plain ITRCZ dispersion at a dose of 10 mg/kg/day i. p.; TAcA/ITRCZ-Cht NPs (*n* = 10), rats received biweekly TAcA and ITRCZ/ITRCZ-Cht NPs (10 mg/kg/day i. p.). At the end of the study, rats were decapitated after administration of a mixture of 87.5 mg/kg ketamine and 12.5 mg/kg xylazine for anesthesia. This was followed by liver tissue harvesting.

### 2.10 Histopathological evaluation and immunohistochemical staining of liver tissue

Standard H&E staining procedures were applied and the score of necroinflammation was assessed as described by Krishna (2021). Additionally, Sirius red staining was performed to evaluate the degree of collagen deposition and to assess the fibrotic area % (Malkusch et al., 1995). Furthermore, Thermo Fisher Scientific (Rockford, IL, United States) supplied rabbit polyclonal antibodies for IHC staining of α-SMA or nuclear factor kappa B (NFκB) p65 subunit (1:800 dilution). In this procedure, we utilized the peroxidase-labeled streptavidin-biotin technology. The liver expression of α-SMA was assessed by measuring the % of the positive area in relation to the entire area and the expression of the p65 subunit was measured by determining the immunopositive cells in 1,000 counted cells. 10 high-power fields were examined for each rat. The α-SMA area% and the fibrosis area % were determined by ImageJ 1.53p software.

### 2.11 Assessment of liver function enzymes and oxidative stress markers

Serum activities of alanine aminotransferase (ALT), aspartate aminotransferase (AST), alkaline phosphatase (ALP), and gamma-glutamyl transferase (γGT) as well as the levels of oxidative stress markers malondialdehyde (MDA), reduced glutathione (GSH), superoxide dismutase (SOD), and catalase (CAT) were determined using assay kits that were obtained from Bio-diagnostic (Giza, Egypt) following the manufacturer’s instructions. All assays were performed in duplicate.

### 2.12 Assessment of inflammation and fibrosis markers

The levels of tumor necrosis factor-alpha (TNF-α), TGF-β, and tissue inhibitor of metalloproteinase-1 (TIMP-1) were measured utilizing kits that were supplied by CUSABIO (Wuhan, China), while platelet-derived growth factor-BB (PDGF-BB) levels were assessed by a kit that was obtained from Abcam (Cambridge, MA, United States) according to the guidelines. All measurements were performed in duplicate.

### 2.13 Determination of hydroxyproline content

The liver content of hydroxyproline was measured as previously outlined by Reddy and Enwemeka (1996). At the start, for 30 min, 25 μL of 20% liver tissue homogenate was exposed to hydrolysis in 25 μL of 2N NaOH at 120°C. Afterward, chloramine T solution (450 μL) was brought into the resultant hydrolysate and permitted to react for 25 min at 25°C. Then, Ehrlich’s solution (500 μL) was introduced into the mixture, and then the mixture was incubated at 65°C for an additional 20 min. This was followed by a cooling for 10 min. The produced color was assessed at 550 nm. The assay was performed in triplicate.

### 2.14 Determination of Hedgehog signaling components SMO, GLI1, and GLI2

To determine the levels of SMO, GLI1, and GLI2, commercial kits were obtained from MyBioSource (San Diego, CA, United States), Fine Test (Wuhan Fine Biotech Corp., Wuhan, China), and Cloud-Clone Corp. (Houston, TX, United States), respectively. All assays followed the manufacturer’s protocols and were performed in duplicate.

### 2.15 Statistical analysis

Minitab 21 software was used to perform statistical analysis for the factorial design. We employed multiple regression analysis to assess the response, as defined by the equation: $R=x_{0}+x_{1}\;A+x_{2}\;B+x_{12}\;AB$ (Nasr et al., 2023b). Other results were statistically analyzed using GraphPad Prism 9.5.0 Software. The one-way ANOVA followed by Tukey’s multiple comparison test was utilized to calculate the difference between the mean values in the case of parametric measurements. For the inflammation score, the Kruskal–Wallis test followed by Dunn’s as a *post hoc* test was utilized. Data are presented as the mean ± standard deviation (parametric data) or as the median ± IQR (non-parametric data).

## 3 Results

### 3.1 Design and preparation of ITRCZ-Cht NPs

A Cht concentration of 0.2% w/v, equivalent to 40 mg of Cht, was chosen to establish a 2:1 ratio with the drug amount. This choice aimed at achieving a reasonable EE%, given the fact that a 0.1% Cht concentration resulted in a remarkably low EE of 46%. Subsequently, two levels of the Cht concentration factor were selected, specifically 0.2% and 0.3% w/v. Concerning Na-TPP, the initial ratio between the quantities of Cht and Na-TPP was established at 3:1. However, no NPs were formed, and the medium remained clear, resembling the transparent state observed with the Cht-ITRCZ solution before the addition of Na-TPP. Even upon increasing the Na-TPP concentration to a 1:1 ratio with Cht, the medium continued to exhibit transparency. This outcome might be attributed to the lower pH of the Cht solution (pH = 2.5), which was utilized to obtain a clear homogenous dispersion of ITRCZ. This lower pH necessitated a higher quantity of Na-TPP than usual to attain a high negative charge density, allowing Na-TPP to establish electrostatic crosslinks with Cht. Consequently, two levels of Na-TPP concentration were selected, explicitly 1% and 1.5% w/v. In this concentration range, the medium exhibited an opalescent suspension zone, indicative of the presence of colloidal particles.

### 3.2 Characterization of ITRCZ-Cht NPs

**FIGURE 1:**
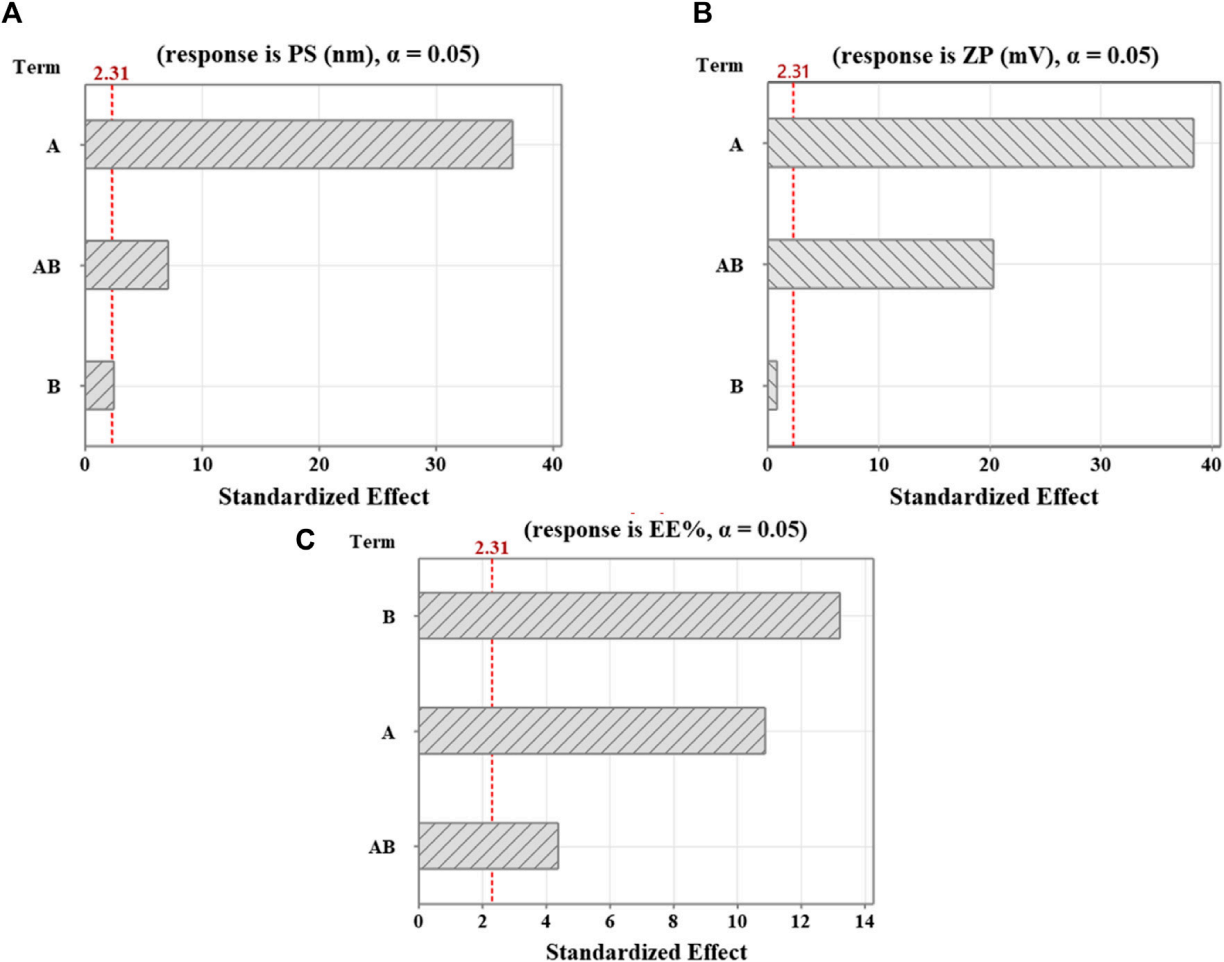
Pareto diagrams illustrating standardized effects on **(A)** PS, **(B)** ZP, and **(C)** EE%. A reference for statistical significance is designated by the red dashed line. The significance level indicated by α = 0.05. A and B factors correspond to Cht and Na-TPP concentration, respectively. AB denotes interaction terms for these factors.

Table 3 presents a summary of the characterization outcomes for the ITRCZ-Cht NPs. The PS of the prepared formulations varied from 169.6 ± 1.77 to 221.13 ± 2.5 nm, demonstrating a consistently narrow size distribution, as reflected by PDI values ≤0.3. The regression equation describing the influence of different factors on PS is expressed as follows: PS=196.808+21.575 A − 1.442 B+4.192 AB . According to the regression analysis and the Pareto chart (Figure 1), all factors exhibited a significant impact on PS (*p-value* < 0.05). The most influential factor was found to be the concentration of Cht (+A). The positive coefficient value suggests that increasing Cht concentration significantly increases PS, potentially due to a rise in polymer particles generating each vesicle, given the established direct relationship between the number of particles and vesicle size (El-Gizawy et al., 2020). The second and third influential factors were represented by (+AB) and (−B), signifying that a rise in Na-TPP concentration resulted in a reduction in PS. This can be attributed to the heightened Na-TPP concentration, resulting in more cross-linkages with the Cht polymer and more compact particles with a reduced hydrodynamic diameter.

**TABLE 3:** Summary of characterization results (mean ± SD, n = 3) for ITRCZ-Cht NPs obtained through the Implemented 2^2^ factorial design.

Formulation	EE (%)	ZP (mV)	PS (nm)	PDI
F1	80.83 ± 1.26	11.56 ± 0.41	180.85 ± 1.80	0.155 ± 0.027
F2	77.58 ± 0.76	24.7 ± 0.30	215.63 ± 2.02	0.261 ± 0.031
F3	89.65 ± 0.57	15.93 ± 0.21	169.6 ± 1.77	0.195 ± 0.017
F4	81.94 ± 0.64	19.97 ± 0.55	221.13 ± 2.50	0.348 ± 0.025

Concerning the surface charge, the ZP values for the formulations ranged from 11.56 ± 0.41 to 24.7 ± 0.30 mV, exhibiting positively charged surfaces attributed to the occurrence of free amino groups in Cht. That positive charge aligns with the study’s objective, as hepatocytes show a heightened affinity for positively charged NPs (Cheng et al., 2012), while NPs are phagocytosed by Kupffer cells in the case of being negatively charged (Poelstra et al., 2012). The regression equation capturing the influence of various factors on ZP is expressed as follows: ZP=18.042+4.292 A − 0.092 B− 2.257 AB . As per the regression equation and the Pareto chart (Figure 1B), all factors significantly impact ZP (*p-value* < 0.05) except for factor B (Na-TPP concentration). The most influential factor was Cht concentration (+A), signifying that an escalation in polymer concentration led to an increase in the ZP value, as expected, given the rise in positive free amino groups with higher Cht concentration. The second factor, represented by (−AB), indicated that concurrently decreasing Cht and increasing Na-TPP concentrations lowered the value of ZP. This effect might be ascribed to a rise in negatively charged phosphate ions accompanying higher Na-TPP concentrations, reacting with amino groups of chitosan which are positively charged and leading to a reduction of the positive charge.

**FIGURE 2:**
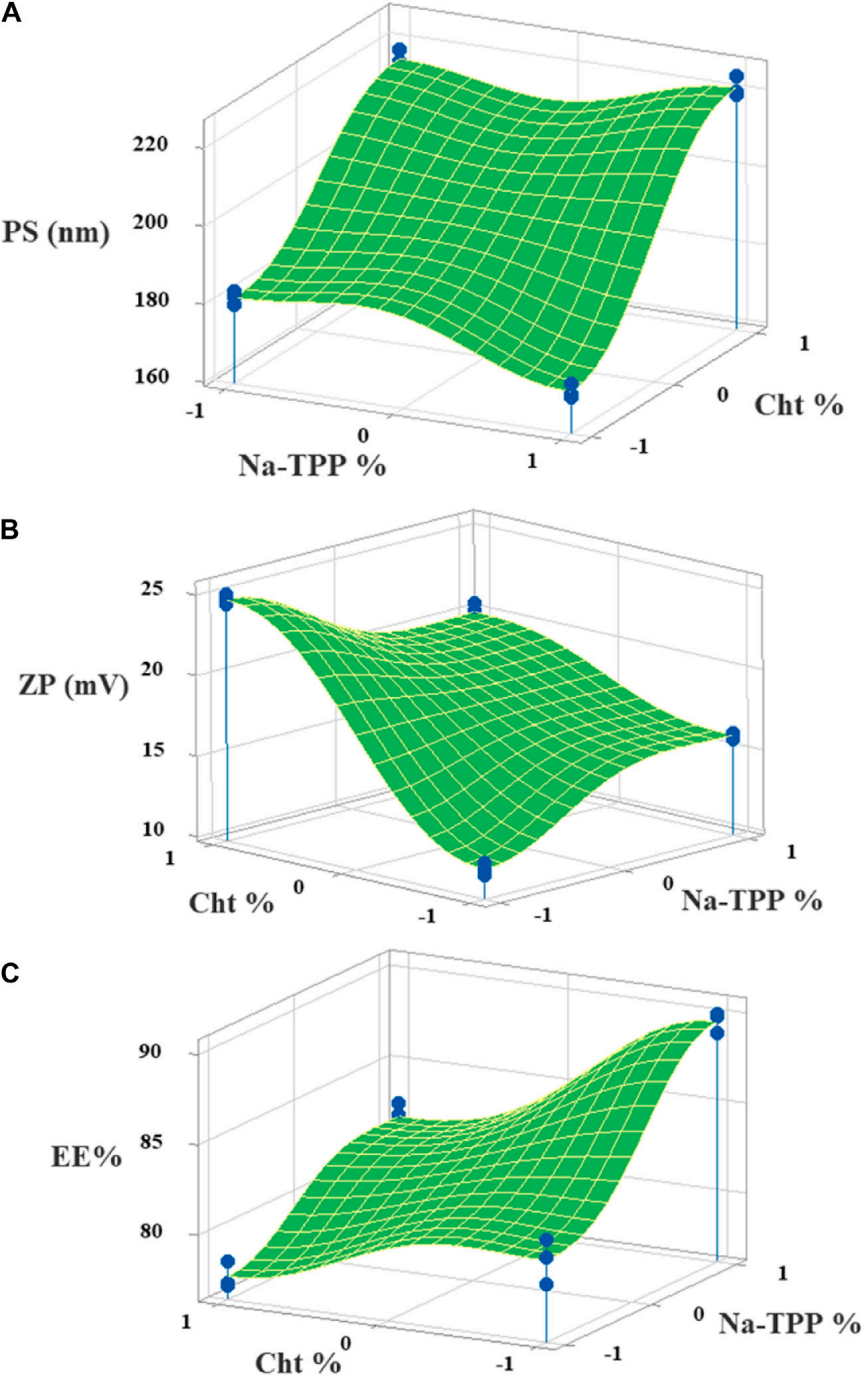
The 3D surface plots of the **(A)** PS, **(B)** ZP, and **(C)** EE% with variances of Cht and Na-TPP concentrations.

Regarding the EE%, the formulations exhibited a range from 77.58% ± 0.76% to 89.65% ± 0.57%. The regression equation detailing the effect of various factors on EE% is as follows: EE%=82.517 − 2.722 A+3.311 B− 1.096 AB . As per the regression equation and the Pareto chart (Figure 1C), all factors significantly influence EE% (*p-value* < 0.05). The most significant factor was Na-TPP concentration (+B), denoting that an increase in Na-TPP concentration led to a rise in EE%. This can be attributed to the elevated Na-TPP concentration fostering more cross-linkages with the Cht polymer, resulting in more compact particles that prevent drug leakage from the NPs’ core. The second factor, denoted by (−A), suggested an inverse relationship between Cht concentration and EE%. This could be explained by higher Cht concentrations increasing the viscosity of the gelation medium, making it more challenging for the drug to enter the NP’s core. Figure 2 displays 3D surface plots visually depicting the influence of the factors on the chosen responses.

### 3.3 Optimization of ITRCZ-Cht NPs

**FIGURE 3:**
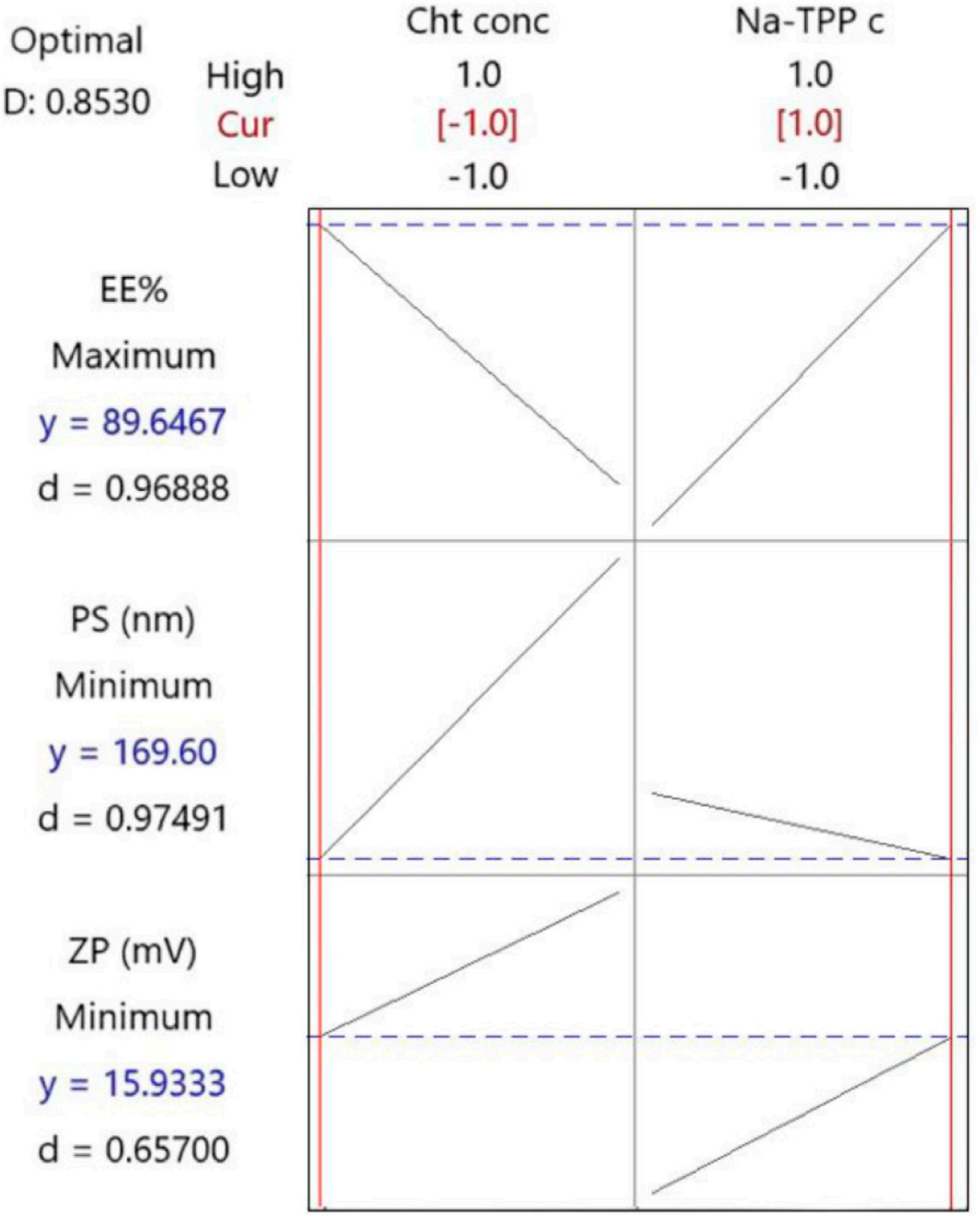
The response optimization plot was created by Minitab. Vertical red lines have been integrated to highlight the existing factor settings. The red numerical values positioned above each column designate the current level settings for the factors. Moreover, horizontal dashed lines along with blue numerical values indicate the responses associated with the present factor levels.

Following the implementation of the response optimization approach, the optimization plot (Figure 3) reveals that the formula yielding optimal results across all measured parameters was (F3) with an optimal desirability of 0.853. F3 contains a Cht concentration of 0.2% w/v and a Na-TPP concentration of 1.5% w/v.

### 3.4 Characterization of the optimized formulation

**FIGURE 4:**
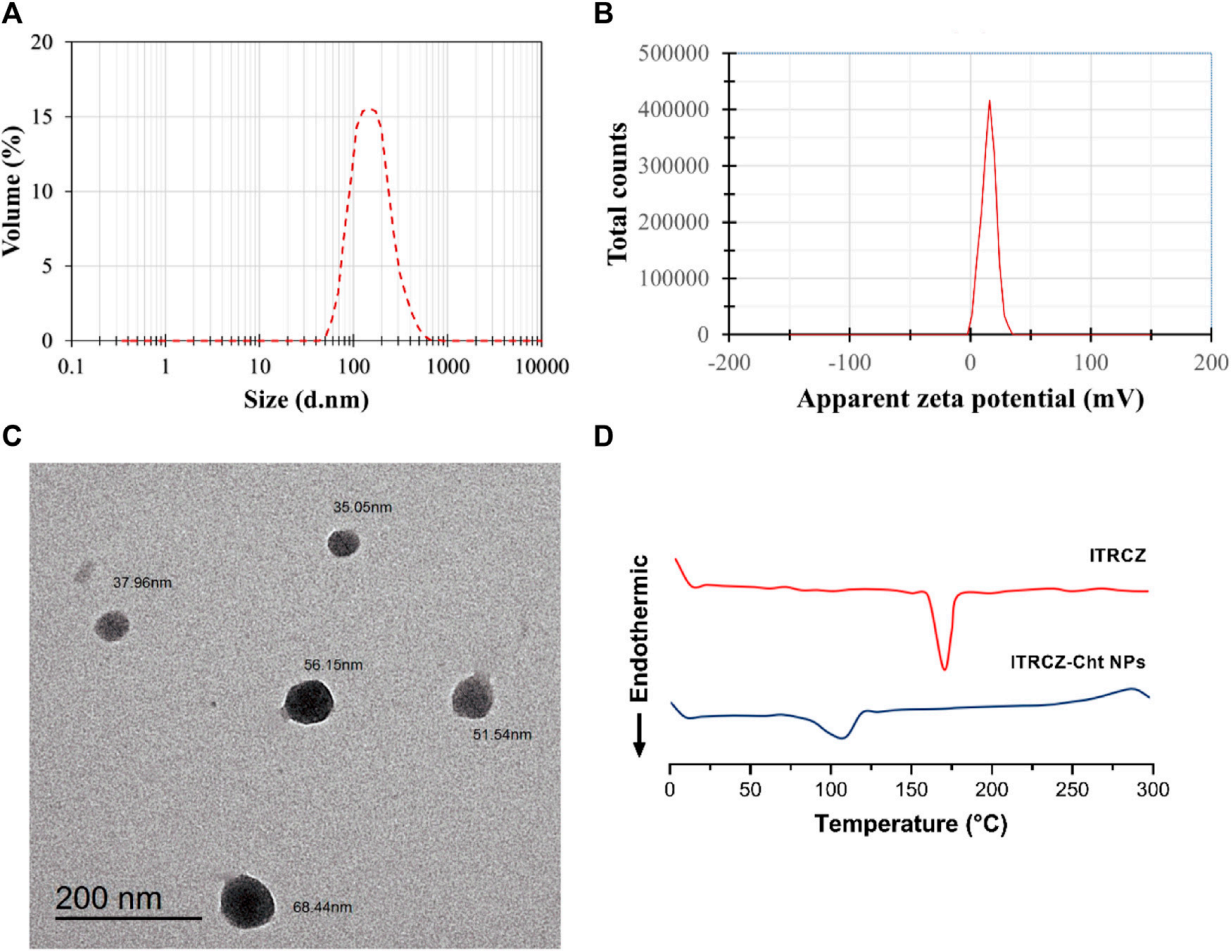
Characterization of the optimized formulation: **(A)** Size distribution by volume, **(B)** zeta potential distribution, **(C)** TEM photograph, and **(D)** DSC thermograms of ITRCZ and lyophilized ITRCZ-Cht NPs.

The optimized formulation (F3) exhibits an EE% of 89.65% ± 0.57%, an LD% of 17.93% ± 0.11%, a ZP of 15.93 ± 0.21 mV (Figure 4A), and a PS of 169.6 ± 1.77 nm (Figure 4B). TEM analysis (Figure 4C) reveals that the optimized ITRCZ-Cht NPs are nanometer-sized, with a narrow size distribution ranging from 35 to 68 nm, and closely spherical in their shape. It is important to note that the PS measured using the DLS analysis is greater than that acquired from TEM micrographs. The method of dynamic light scattering calculates the hydrodynamic particle diameter using a multi-angle measuring approach, while the TEM experiment provides the actual particle diameter (Alshawwa et al., 2023).

The optimized formulation underwent further characterization concerning its thermal properties utilizing DSC. Figure 4D illustrates the thermal behavior of the ITRCZ, indicating a distinctive sharp endothermic peak at 169.9°C. This peak is associated with the drug’s melting point and crystalline nature. Meanwhile, the thermogram of the optimized ITRCZ-loaded Cht NPs reveals an endothermic peak observed within the temperature range of 85°C–120°C. This particular peak is ascribed to the evaporation of absorbed water. The DSC outcomes provide compelling evidence of the successful entrapment of ITRCZ within the Cht NPs. This is evidenced by the absence of the characteristic melting peak of ITRCZ in the formulation of ITRCZ-Cht NPs, signifying the presence of ITRCZ in a non-crystalline state.

**FIGURE 5:**
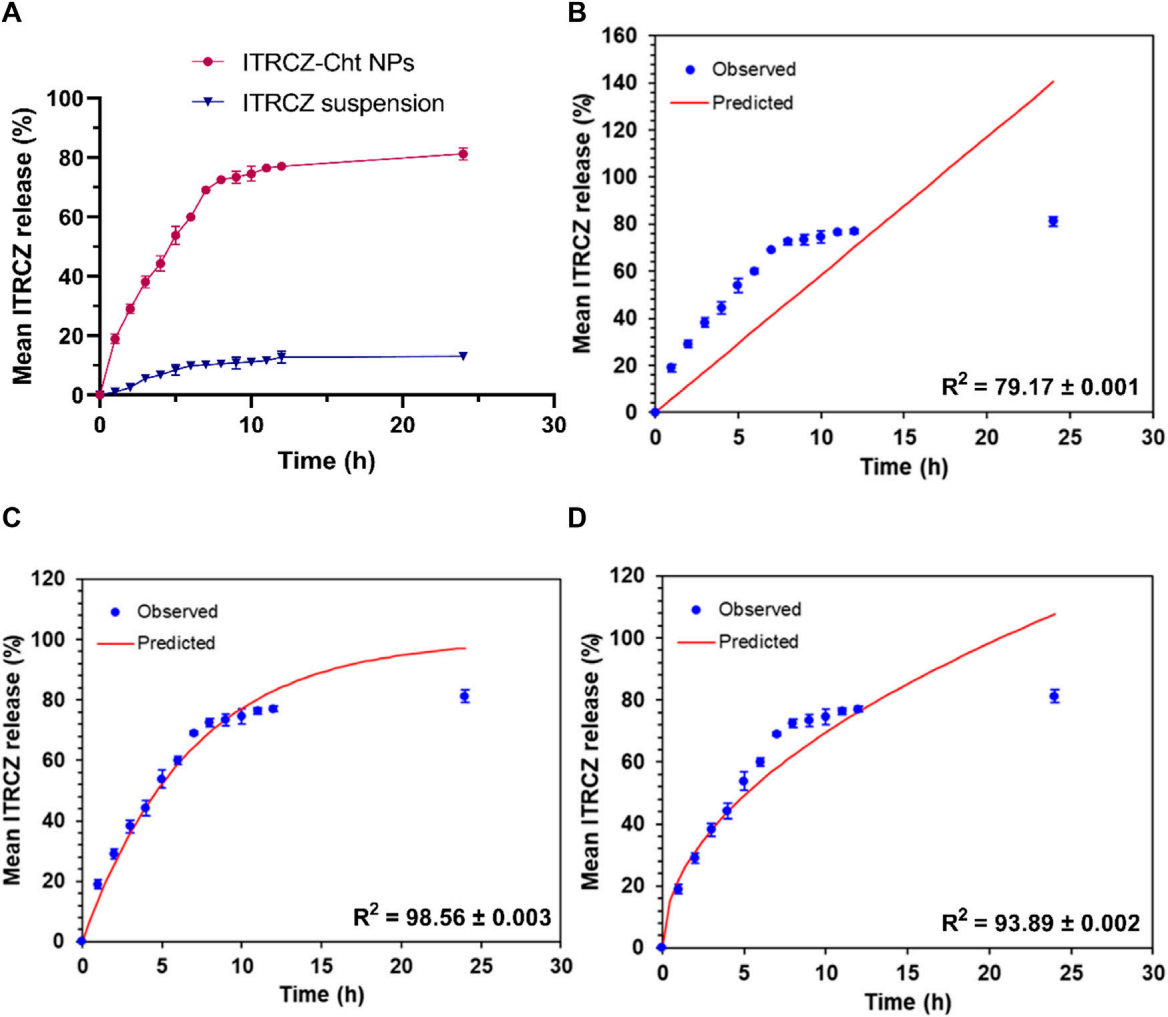
*In vitro* release study: **(A)** Mean cumulative release% of ITRCZ (±SD, n = 3) from ITRCZ-Cht NPs and ITRCZ suspension in PBS at pH 7.4. Panels **(B)**, **(C)**, and **(D)** illustrate the *in vitro* release kinetics for ITRCZ-Cht NPs, with each panel displaying the mean cumulative release percentage fitted to the zero-order, first-order, and Higuchi release models, respectively.

Furthermore, a comparative investigation into the release profiles of ITRCZ suspension and ITRCZ-Cht NPs was conducted over 24 h in PBS at pH 7.4, as depicted in Figure 5A. The observed mean cumulative release percentages were 81.23% ± 2.08% and 13.17% ± 1.12% for ITRCZ-Cht NPs and ITRCZ suspension, respectively. The significant increase (*p-value* < 0.05) in cumulative release from Cht NPs compared to the plain ITRCZ suspension can be attributed to the nano-sized particles developed in this formulation. The heightened surface area associated with smaller particle sizes facilitates increased drug retention, resulting in enhanced ITRCZ solubilization and subsequent elevation in drug release. Conversely, the lower release observed with ITRCZ suspension aligns with the inherent challenges of this drug’s crystalline and highly poorly soluble nature (Parikh et al., 2016). Examining the release pattern of ITRCZ-Cht NPs reveals a biphasic profile characterized by a first burst within the first 7 h, succeeded by a sustained release. The initial rapid release is attributed to surface-loaded or near-surface-encapsulated drug molecules, allowing facile diffusion from the NPs’ matrix into the surrounding medium during the initial incubation period. Upon fitting the release data of ITRCZ-Cht NPs to zero-order (Figure 5B), first-order (Figure 5C), and Higuchi models (Figure 5D), the highest *R* ^*2*^ value was obtained for the Higuchi model (93.89 ± 0.002). Additionally, the *n* exponent value of the Korsmeyer-Peppas model was determined to be 0.38 ± 0.011, indicating that the release of ITRCZ from Cht NPs follows Fickian diffusion. This implies that the primary mechanism governing drug release is diffusion-controlled.

**FIGURE 6:**
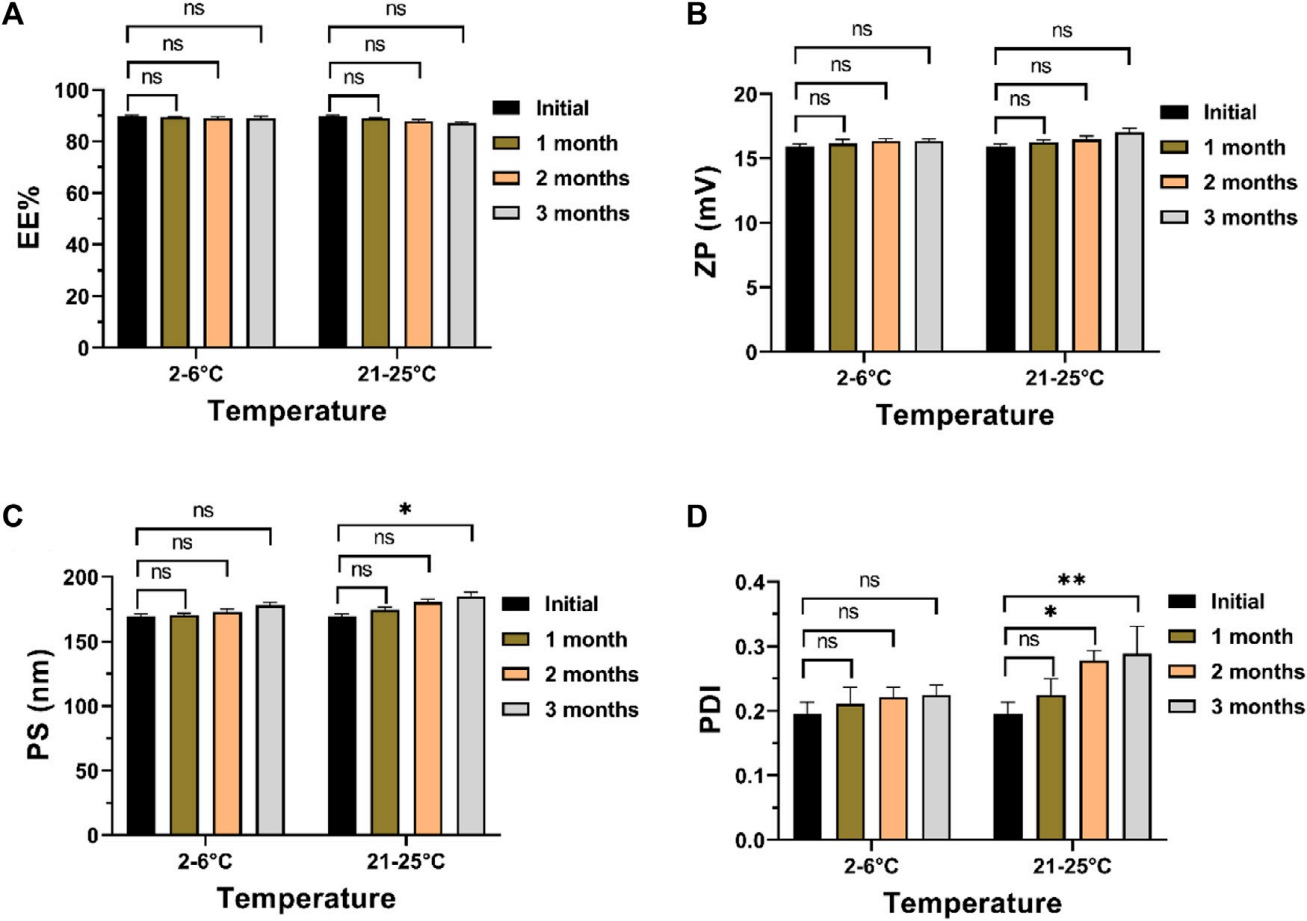
Physical stability assessment of the optimized formulation after 3 months of storage at 2°C–6°C or 21°C–52°C: **(A)** EE%, **(B)** ZP, **(C)** PS, and **(D)** PDI. Data presented as mean ± SD, *n* = 3.

In examining the impact of storage conditions on the physical stability of the optimized formula, a marginal augmentation in PS and PDI was observed concurrently with a decline in ZP and EE% (Figure 6). The decline in EE% is posited to arise from the structural compromise of the chitosan matrix, resulting in the expulsion of ITRCZ from Loaded Cht NPs. The inherent swelling characteristics and moisture adsorption propensity of chitosan within the storage environment may have induced matrix degradation (Szymańska and Winnicka, 2015). Consequently, diminished overall surface charges, decreased inter-particle repulsion, and increased particle aggregation ensued, leading to reduced ZP values and elevated PDI values indicative of a broader distribution of NPs’ size. Notably, these alterations exhibited no statistical significance (*p-value* > 0.05) following a 3-month storage duration at refrigerated conditions (2°C–6°C). Conversely, a noteworthy increase in PDI and PS values was evident at room temperature after 2 and 3 months of storage, respectively. Consequently, storing the optimized formulation under refrigerated conditions is advisable to enhance stability and mitigate nanoparticle aggregation.

### 3.5 Liver partition coefficient (Kp, liver) determination

**FIGURE 7:**
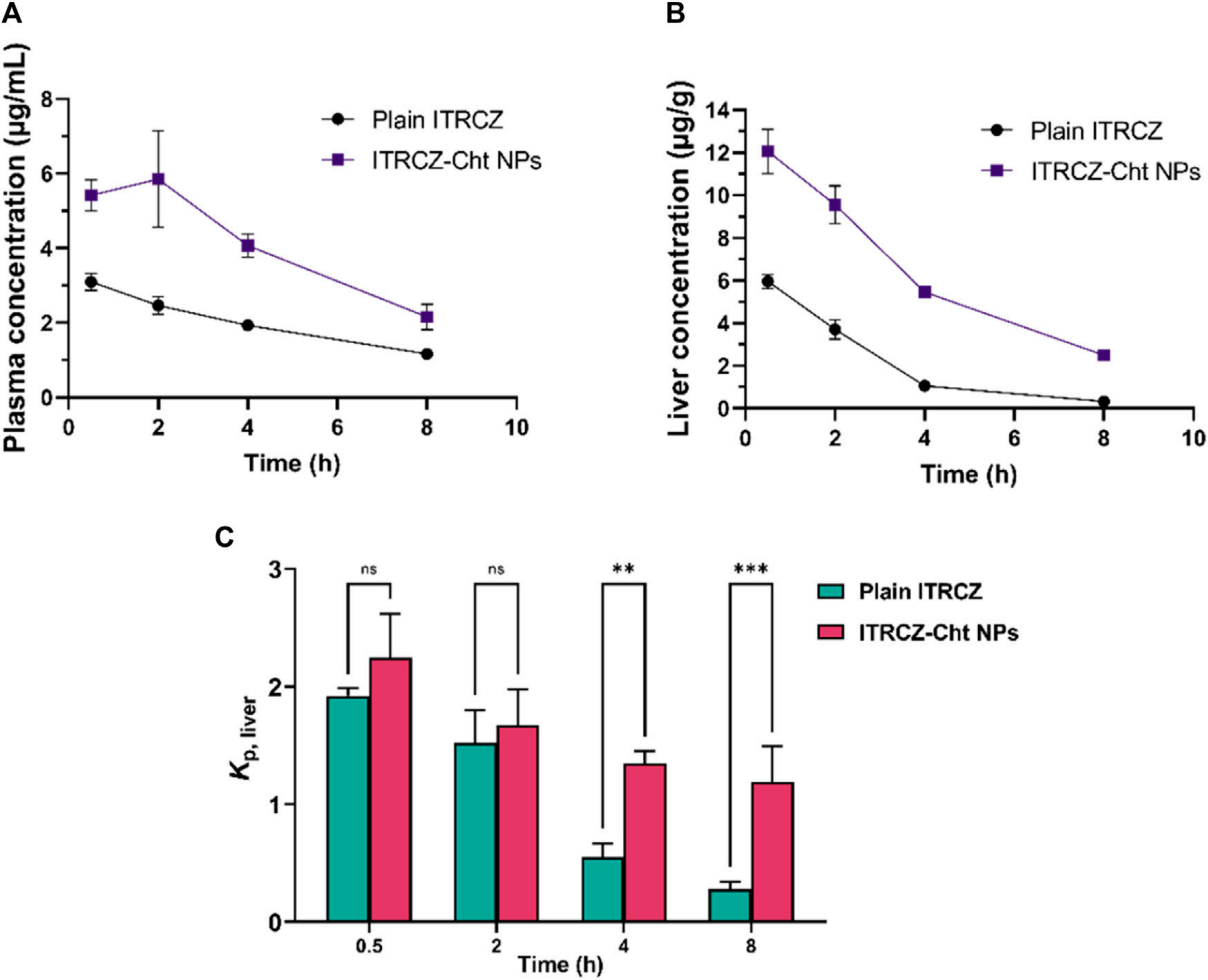
The mean plasma concentration **(A)**, liver concentration **(B)**, and liver partition coefficient (Kp, liver) **(C)** of ITRCZ (±SD, *n* = 3) after the administration of a single intraperitoneal dose of plain ITRCZ and ITRCZ-Cht NPs in a dose equivalent to 10 mg/kg of ITRCZ into male Sprague Dawley rats. The significance level between pairs is indicated by the number of symbols, **p < 0.01, ***p < 0.001, and ns = non-significant.

Figure 7 illustrates the mean liver partition coefficient (*K*_*p, liver*_) of ITRCZ after intraperitoneal administration of plain ITRCZ and ITRCZ-Cht NPs at a dose equivalent to 10 mg/kg of ITRCZ. ITRCZ-Cht NPs showed a relatively higher *K*_*p, liver*_ at 0.5- and 2 h after injection. However, this difference was not statistically significant when compared to plain ITRCZ. On the other hand, compared to plain ITRCZ, there was a notable increase in the *K*_*p, liver*_ value of ITRCZ-Cht NPs at 4 and 8 h. Given that ITRCZ travels through multiple pathways for primary metabolism in the liver, the decreased *K*_*p,*__*and*_ _*liver*_ value for plain ITRCZ could be explained by the increased accessibility of the unbound drug to its metabolizing enzymes (Willems et al., 2001). Conversely, when ITRCZ was administered using Cht NPs, the liver value increased *K*_*p, liver*_. This could be because the liver has a higher quantity of ITRCZ, which lowers the rate of enzymatic metabolism because of enzyme activity saturation (Shin et al., 2004). Moreover, the NPs’ significant systemic absorption from the peritoneal cavity and the manufactured NPs’ physicochemical characteristics, which included a positively charged surface or a reduced particle size, enabled the NPs to accumulate in the liver via a passively targeted pathway. The results highlight how effective Cht NPs are at increasing the amount of ITRCZ that accumulates in liver tissues, which increases the drug’s potential for treating liver fibrosis.

### 3.6 Effect of ITRCZ-Cht NPs on primary rat hepatocyte viability and LDH activity

**FIGURE 8:**
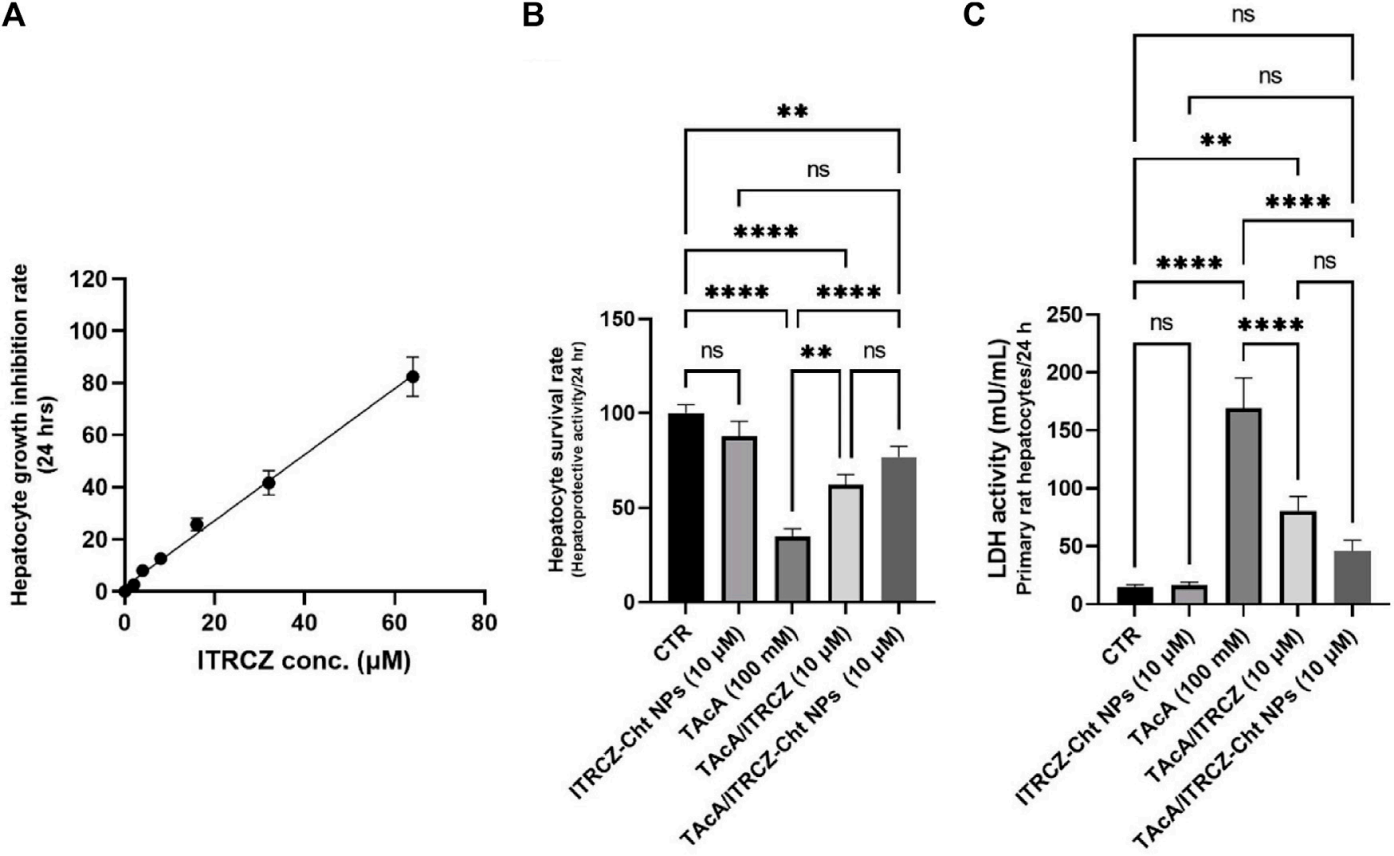
Effect of ITRCZ on hepatocyte growth inhibition rate after 24 h incubation with isolated rat hepatocytes **(A)**; effect of ITRCZ-Cht NPs on hepatocyte survival rate after 24 h of incubation with isolated primary rat hepatocytes **(B)**; effect of ITRCZ-Cht NPs on LDH activity **(C)**. The significance level is denoted by paired comparisons. **, *p* < 0.01; ****, *p* < 0.0001.

Figure 8A displays the hepatocyte growth inhibition rate in response to plain ITRCZ dispersion and signifies that at a dose of 10 µM of ITRCZ, the growth inhibition rate was approximately 15%. Therefore, the 10 µM dose was chosen to be safe on primary rat hepatocytes and was selected for investigating its potential protective effect in the isolated primary rat hepatocytes exposed to 100 mM of TAcA. As demonstrated in Figure 8B, TAcA treatment at the mentioned dose caused a significant decrease in hepatocyte survival alongside a remarkable increase in LDH activity (Figure 8C).

However, treatment with plain ITRCZ (at a dose of 10 µM) successfully mitigated this noxious effect as indicated by the profound increase in hepatocyte survival (*p*-value < 0.01) as well as a significant decline in LDH activity (*p*-value < 0.0001) compared with TAcA group. On the other hand, treatment with ITRCZ-Cht NPs exhibited a remarkably significant increase in the hepatocyte survival rate (*p*-value < 0.0001) and a notable reduction in LDH activity (*p*-value < 0.0001) when compared with those of the TAcA group. Furthermore, ITRCZ-Cht NPs exhibited a strong trend towards significance compared to plain ITRCZ regarding hepatocyte survival rate (*p* = 0.062) and LDH activity (*p* = 0.064). These encouraging findings prompted us to initiate *in vivo* investigations.

### 3.7 ITRCZ alleviated TAcA-induced histopathological alterations in liver tissue

**FIGURE 9:**
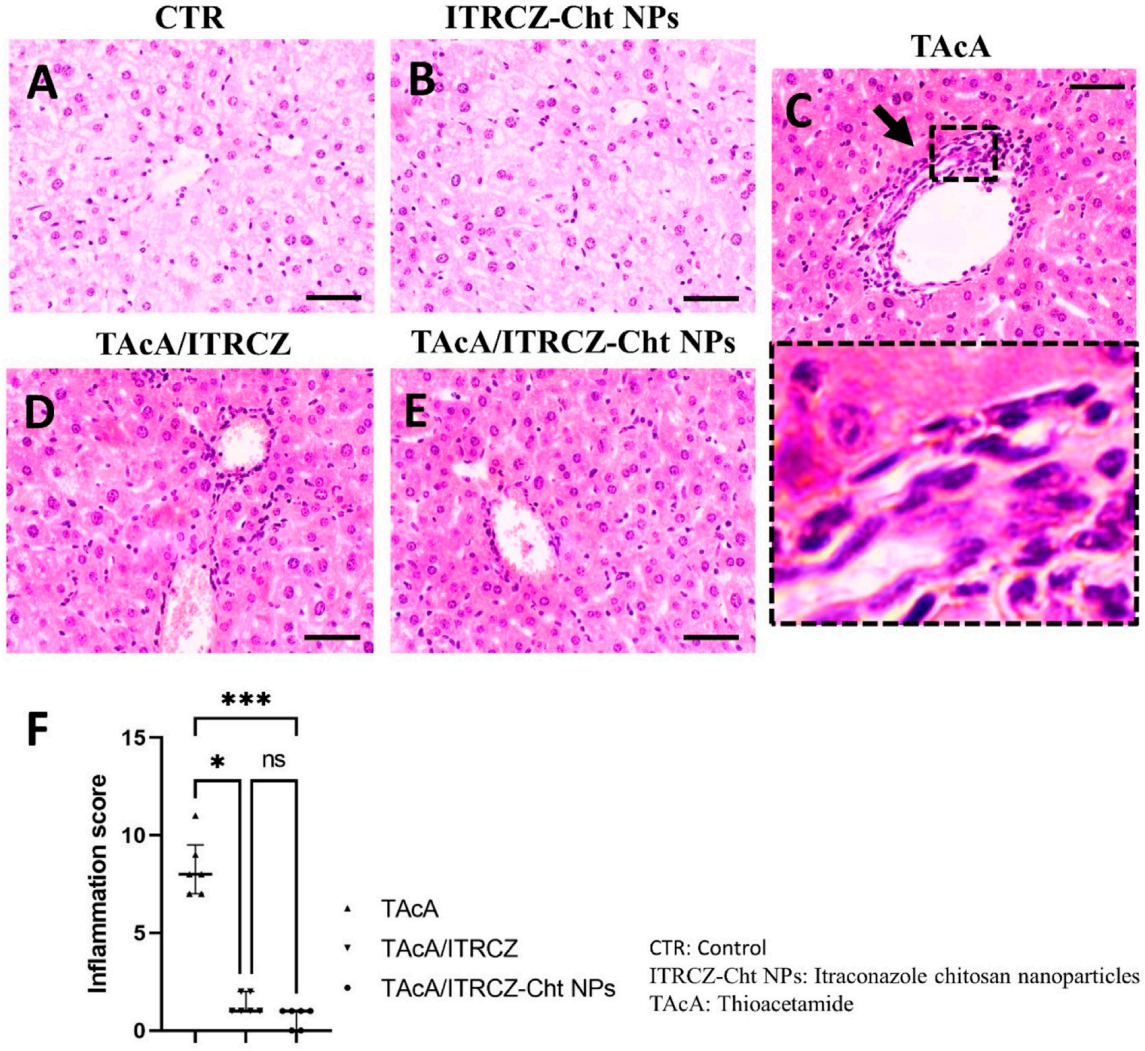
Effect of different treatments on liver sections stained with H&E stain. Arrows indicate inflammatory cell infiltration. F panel illustrates the inflammation score. Scale bar = 50 µm. The significance level is denoted by paired comparisons. *, *p* < 0.05; ***, *p* < 0.001.

**FIGURE 10:**
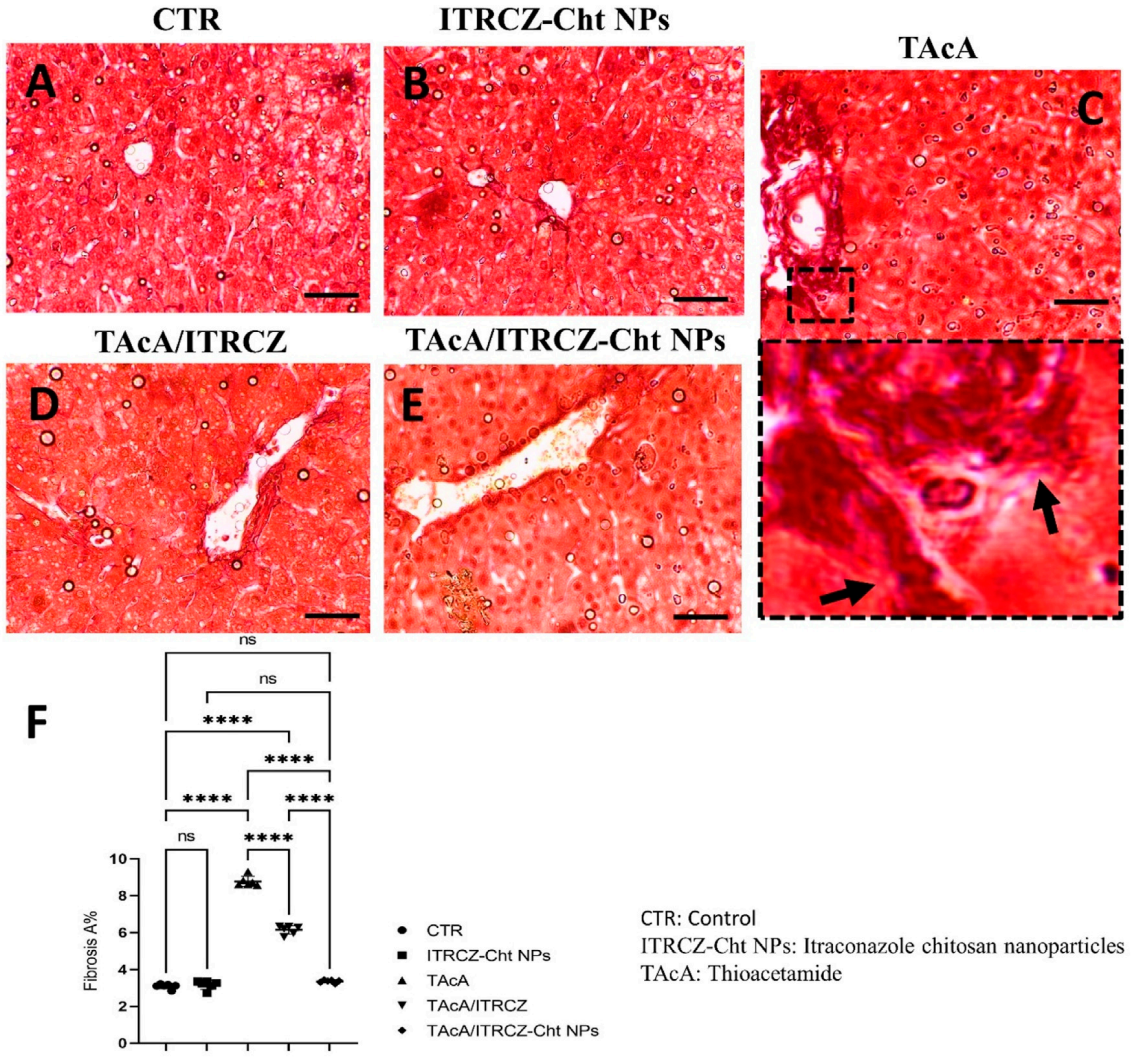
Effect of different treatments on liver sections stained with Sirius red stain. Arrows indicate collagen deposition. F panel illustrates fibrosis area %. Scale bar = 50 µm. The significance level is denoted by paired comparisons. ****, *p* < 0.0001.

**FIGURE 11:**
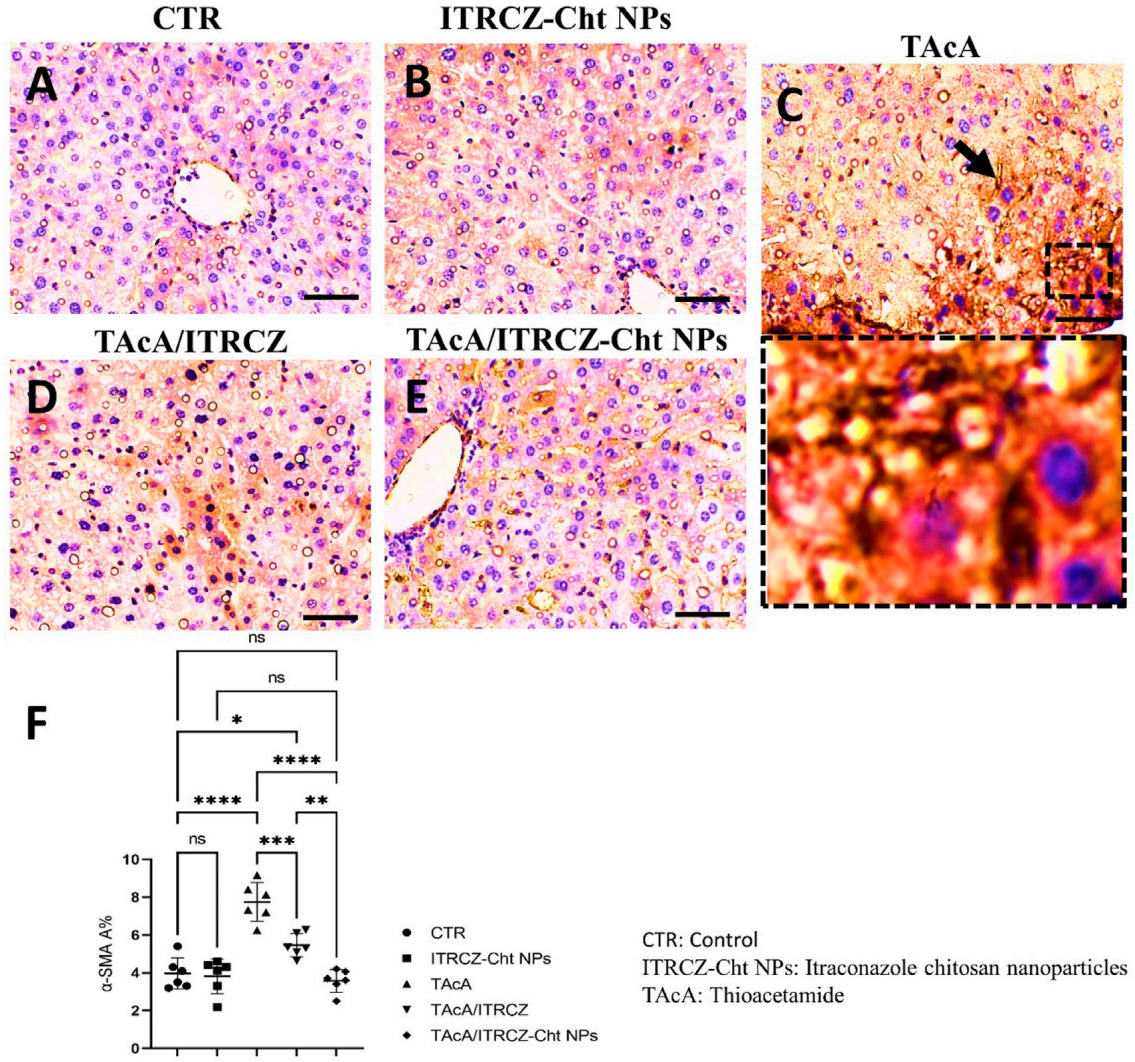
Effect of different treatments on liver sections immunostained with α-SMA antibodies. The arrow indicates increased tissue expression of α-SMA. F panel illustrates α-SMA area %. Scale bar = 50 µm. The significance level is denoted by paired comparisons. *, *p* < 0.05; **, *p* < 0.01; ***, *p* < 0.001; ****, *p* < 0.0001.

**FIGURE 12:**
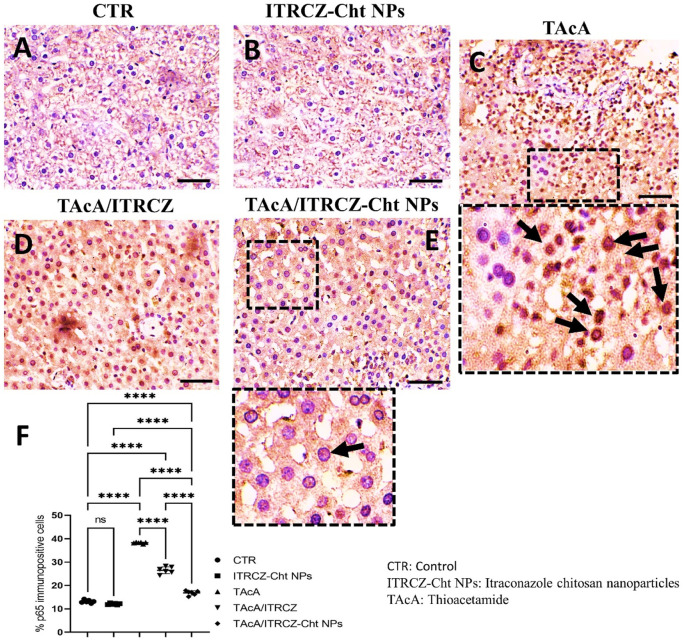
Effect of different treatments on liver sections immunostained with p65 antibodies. Arrows in C indicate nuclear translocation of p65 subunits (positively stained nuclei). Arrow in E indicates non-nuclear translocation of p65 (unstained nuclei). F panel illustrates % p65 immunopositive cells. Scale bar = 50 µm. The significance level is denoted by paired comparisons. ****, *p* < 0.0001.

As depicted, exposure of rats to TAcA resulted in inflammatory (Figure 9) and fibrotic changes (Figure 10) in the liver microstructure. Additionally, TAcA resulted in the increased immunoexpression of the fibrosis marker α-SMA (Figure 11) and nuclear localization of the inflammatory marker p65 as indicated by nuclear brown staining (Figure 12). Furthermore, results of the present work showed that TAcA exposure caused a significant rise in necroinflammation score (Figure 9F), fibrosis area % (Figure 10F), α-SMA area % (Figure 11F), and p65% of immunopositive cells (Figure 12F) compared to control rats. Treatment with plain ITRCZ and more pronouncedly ITRCZ-Cht NPs significantly decreased these levels with respect to TAcA-exposed rats.

### 3.8 ITRCZ modified TAcA-provoked changes in the serum levels of ALT, AST, ALP, and γGT serum activity

**FIGURE 13:**
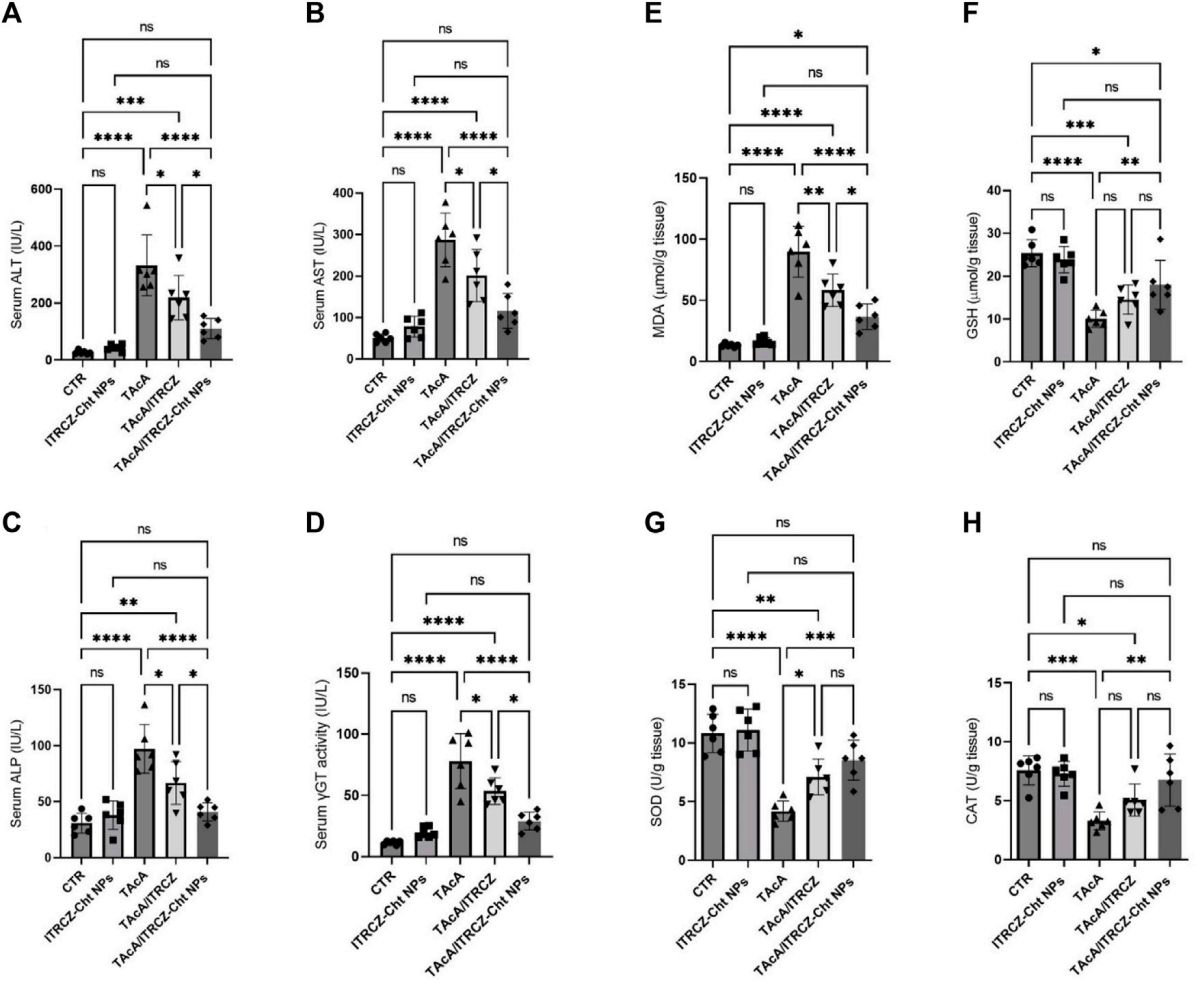
Effect of plain ITRCZ dispersion and ITRCZ-Cht NPs on the serum activities of ALT **(A)**, AST **(B)**, ALP **(C)**, γGT **(D)**, MDA **(E)**, GSH **(F)**, SOD **(G)**, and CAT **(H)** levels in TAcA-intoxicated rats. The significance level is denoted by paired comparisons. *, *p* < 0.05; **, *p* < 0.01; ***, *p* < 0.001; ****, *p* < 0.0001.

Results illustrated in Figures 13A–D demonstrated that TAcA exposure significantly elevated the serum ALT (A), AST (B), ALP (C), and γGT (D) activities, compared to the control group. Meanwhile, plain ITRCZ treatment of TAcA-intoxicated animals profoundly dampened the reported elevation in comparison to the TAcA-exposed group. On the other hand, treatment with ITRCZ-Cht NPs demonstrated a significant reduction in the enzyme serum activities compared to those reported with the plain ITRCZ dispersion.

### 3.9 Effect on TAcA-provoked changes in MDA and GSH serum levels as well as SOD and CAT activities

As depicted in Figures 13E–H, hepatic tissue from TAcA-treated rats displayed significant elevation in MDA (E) serum level along with a remarkable decrease in GSH (F) levels and the activity of each SOD (G) and CAT (H), concerning control animals. Conversely, these pro-oxidant effects in TAcA-intoxicated rats were modulated by ITRCZ treatment as reflected by the significant decline in MDA level as well as a significant increase in SOD activity, in comparison to the TAcA group. However, GSH level and CAT activity demonstrated a statistically insignificant increase in the TAcA/ITRCZ rat group. On the other hand, treatment with ITRCZ-Cht NPs demonstrated a significant decrease in the levels of MDA compared with those reported with the TAcA group. Additionally, ITRCZ-Cht NPs demonstrated a significant decrease in the levels of MDA and a significant increase in GSH levels and the activities of SOD and CAT in comparison with those reported in the TAcA group. In this context, ITRCZ-Cht NPs demonstrated outperformance over plain ITRCZ dispersion in decreasing MDA levels.

### 3.10 ITRCZ-Cht NPs reversed TAcA-induced alterations in TNF-α, TGF-β, PDGF-BB TIMP-1, and hydroxyproline liver content

**FIGURE 14:**
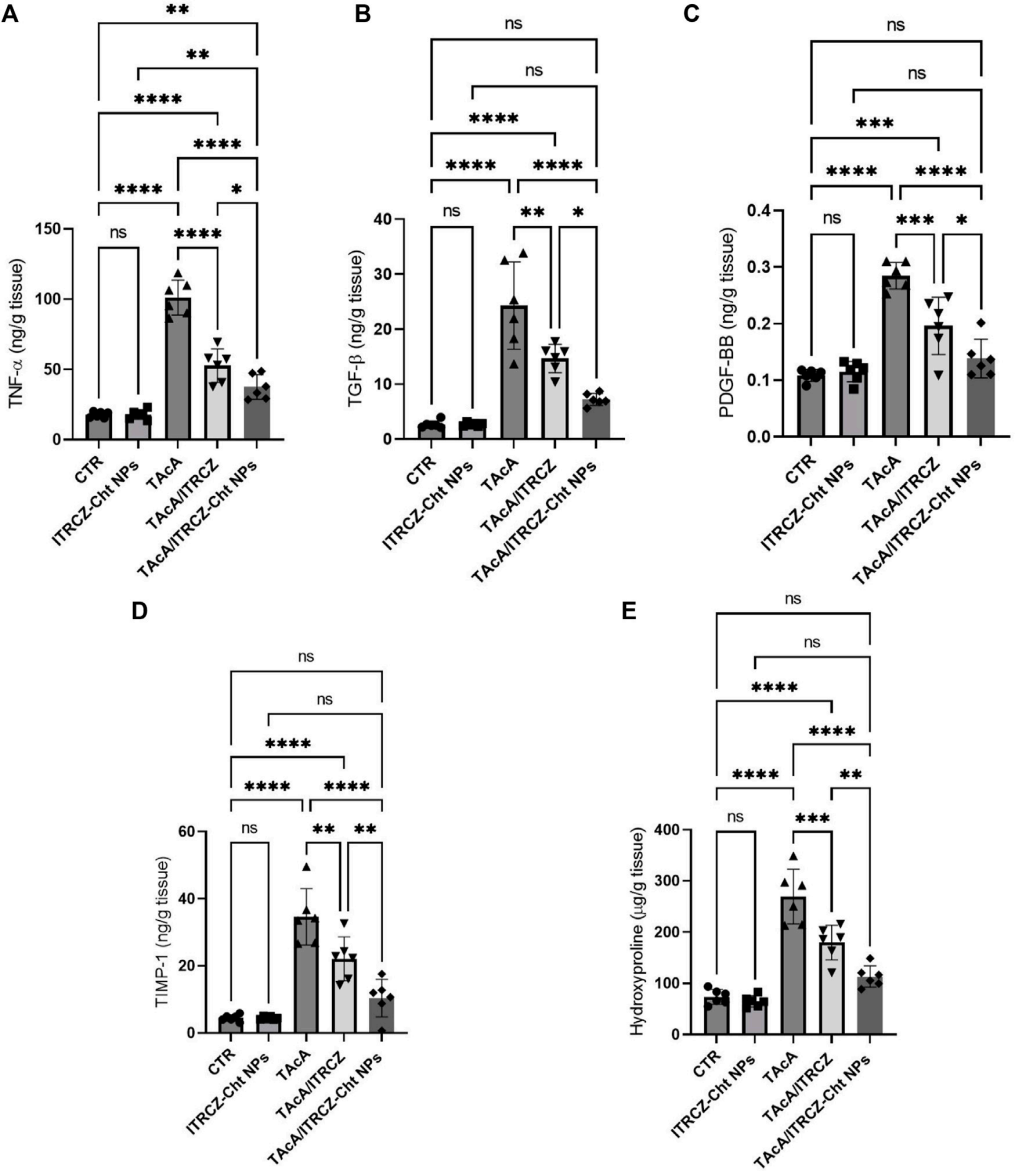
Effect of plain ITRCZ dispersion and ITRCZ-Cht NPs on the levels of TNF-α **(A)**, TGF-β **(B)**, PDGF-BB **(C)**, TIMP-1 **(D)** and hydroxyproline **(E)** in TAcA-intoxicated rats. The significance level is denoted by paired comparisons. *, *p* < 0.05; **, *p* < 0.01; ***, *p* < 0.001; ****, *p* < 0.0001.

In rats intoxicated with TAcA, hepatic protein levels of TNF-α, TGF-β, PDGF-BB, TIMP-1, and hydroxyproline demonstrated a statistically significant increase, compared to control animals as shown in Figure 14A–E, respectively. Notably, plain ITRCZ treatment of TAcA-exposed rats significantly downregulated hepatic protein expression of aforementioned parameters, with respect to the TAcA-treated group. In this regard, ITRCZ-Cht NPs surpassed plain ITRCZ dispersion in decreasing these parameters and displayed significant decreases compared with those levels in the TAcA/ITRCZ group of rats.

### 3.11 ITRCZ-Cht NPs attenuated TAcA-elicited changes in hepatic protein expression of SMO, GLI1, and GLI2

**FIGURE 15:**
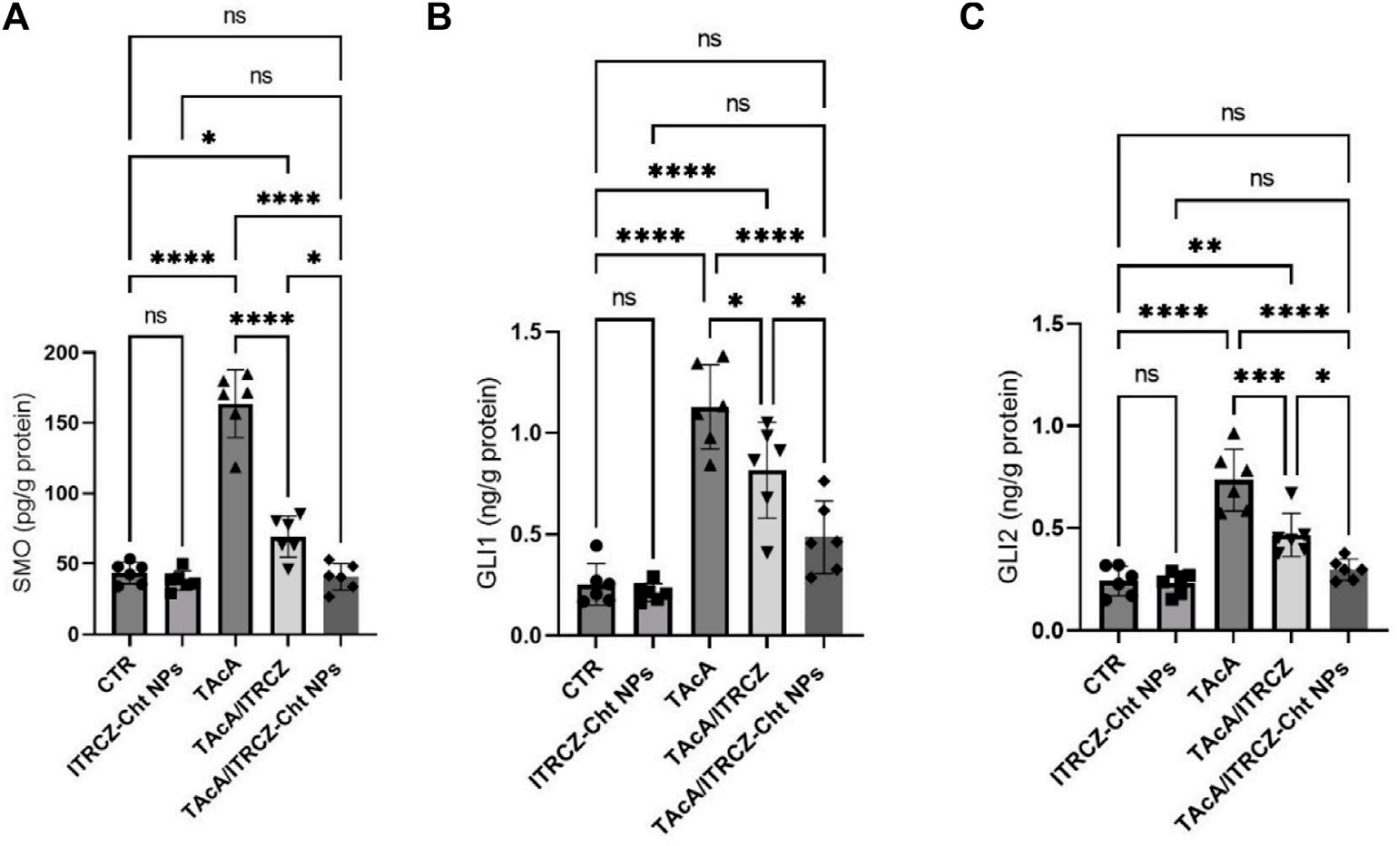
Effect of plain ITRCZ dispersion and ITRCZ-Cht NPs on the levels of SMO **(A)**, GLI1 **(B)**, and GLI2 **(C)** in TAcA-intoxicated rats. The significance level is denoted by paired comparisons. *, *p* < 0.05; **, *p* < 0.01; ***, *p* < 0.001; ****, *p* < 0.0001.

As depicted in Figure 15, the results of the current work demonstrated that TAcA exposure caused a profound increase in the protein expression of Hedgehog signaling components SMO, GLI1, and GLI2 in hepatic tissue compared to control rats. Meanwhile, the reported effect was attenuated by the treatment of TAcA-intoxicated rats with plain ITRCZ as evidenced by the statistically significant decline in hepatic protein expression of the previously stated parameters, in comparison to the TAcA-treated group. Interestingly, ITRCZ-Cht NPs demonstrated a more powerful effect in this regard as indicated by the significant decrease in these Hedgehog components in the TAcA/ITRCZ-Cht NPs group compared with those of the TAcA/ITRCZ group.

## 4 Discussion

Liver fibrosis is a defective wound healing mechanism characterized by distortion of normal hepatic architecture and formation of tissue scarring culminating in liver cirrhosis with consequent impairment of liver function (Dongiovanni et al., 2014). Of note, activated HSCs represent the principal cellular culprits for liver fibrosis (Friedman, 2008). Following HSC activation, mainly due to significant hepatic tissue damage, such cells undergo remarkable trans-differentiation into a contractile fibrogenic collagen-secreting phenotype that is primarily implicated in the pathogenesis of hepatic fibrosis (Bataller and Brenner, 2005; Wallace et al., 2008). Notably, several morphogenic signaling cascades were reported to be aberrantly activated or deregulated in liver fibrosis including the Hedgehog pathway. Herein, the present work examined the effectiveness of ITRCZ-Cht NPs in mitigating hepatic fibrosis by modulating the key fibrogenic Hedgehog components.

The release mechanism of ITRCZ from chitosan NPs in the liver is likely influenced by several factors, including nanoparticle degradation, drug diffusion, and potential interactions with liver tissue components. Additionally, the liver’s unique microenvironment, characterized by enzymatic and macrophage activity, plays a significant role. Based on the *in vitro* release analysis in the present study, it was observed that NPs facilitated a notably increased drug release in comparison to plain ITRCZ. This enhancement could be attributed to the nanostructured characteristics of the particles, which led to augmented plasma concentrations of ITRCZ. The mechanism governing this release involved both diffusion and polymer erosion. Chitosan NPs are known to degrade gradually under physiological conditions (Herdiana et al., 2022), consequently promoting a greater release of encapsulated drugs. Nonetheless, the predominant mode of release was diffusion, as indicated by the n exponent value derived from the Korsmeyer-Peppas model (0.38 ± 0.011). Moreover, the mucoadhesive property of chitosan, which promotes the adherence of drugs to mucosal surfaces and enhances permeability across the biological membranes (Kumar et al., 2016), played a significant role in elevating plasma concentrations. As a result, the heightened uptake of ITRCZ in the liver via NPs can be ascribed to the amplified blood levels attained through these mechanisms. Additionally, the mucoadhesive nature of chitosan prolonged the residence time of NPs in the liver (Sonin et al., 2020). In addition, the optimized formulation exhibited a cationic character, manifesting a positive surface charge of 15.93 ± 0.21 mV. Such a charge profile is conducive to liver targeting, as the surface charge of NPs significantly influences their cellular uptake. It has been documented that hepatocytes exhibit a heightened propensity to internalize positively charged NPs (Xiao et al., 2011; Nasr et al., 2023a). Furthermore, NPs bearing a cationic charge density have been shown to enhance membrane affinity and nucleic acid binding affinity according to existing literature (Brannon-Peppas and Blanchette, 2004; Cheng et al., 2012).

Concerning the distinctive microenvironment of the liver, encompassing enzymatic and macrophage activity, it is noteworthy that the enzyme activity saturation pattern observed with ITRCZ could elucidate the elevated liver accumulation of ITRCZ following administration via NPs. The heightened concentration of ITRCZ within the liver induced by NPs may lead to saturation of metabolic enzymes, thereby reducing the degree of ITRCZ metabolism and consequently enhancing drug concentration within the liver compared to its plain form. Additionally, NPs are renowned for their inherent tendency to accumulate within the macrophage system (Song et al., 2014), with the liver being the primary site of macrophage abundance. This further underscores the augmented drug concentration within the liver subsequent to NPs administration.

In the current study, we investigated the use of ITRCZ-Cht NPs. This formulation functioned to overcome the limitations of ITRCZ (a BCS class II medication). We formulated and optimized Cht NPs loaded with ITRCZ as a new strategy for managing liver fibrosis. ITRCZ-Cht NPs were developed and optimized utilizing a developed 2^2^ full factorial design. The optimized formulation (F3) underwent comprehensive *in vitro* and *in vivo* characterization. *In vitro* assessments revealed that F3 exhibited an entrapment efficiency of 89.65% ± 0.57%, a 169.6 ± 1.77 nm particle size, and a zeta potential of +15.93 ± 0.21 mV. Moreover, *in vitro* release studies indicated that the release of ITRCZ from F3 adhered closely to the first-order model, demonstrating a significant enhancement (*p-value* < 0.05) in cumulative release compared to plain ITRCZ dispersion. This optimized new formulation showed a higher Kp, liver value compared to plain drug reflecting its ability to enhance ITRCZ concentration within liver tissues. Furthermore, this formulation was found to increase the survival rate and decrease the release of LDH in TAcA-intoxicated primary rat hepatocytes.

The current study has presented the first *in vivo* evidence advocating the potential antifibrotic effect of ITRCZ against TAcA-induced liver fibrosis. Notably, ITRCZ-Cht NP administration improved liver function and microstructure and ameliorated TAcA-induced collagen deposition. This observation was supported by the reported suppression of all examined fibrotic markers including hydroxyproline, TGF-β, α-SMA immunoexpression as well as the documented downregulation of TIMP-1 and PDGF-BB protein expressions. This was accompanied by alleviating hepatic inflammation as denoted by decreased TNF-α protein levels and diminished nuclear translocation of the inflammation marker p65. Moreover, these findings were confirmed by the reported reduction in the necroinflammation score. Moreover, ITRCZ-Cht NPs displayed promise against TAcA-induced oxidative stress.

Remarkably, the role of oxidative stress in the development and progression of liver fibrosis is well established where oxidative stress has been identified as a remarkable feature of experimental models of liver fibrosis (Sánchez-Valle et al., 2012). Initially, oxidative stress and ROS generation induce hepatocyte necrosis and apoptosis consequent to the destruction of cellular components including lipids, proteins and DNA leading to the intensification of inflammatory responses and initiation of fibrogenic events (Heeba and Mahmoud, 2014; Li et al., 2016). Moreover, ROS generation stimulates inflammatory signaling culminating in hepatic inflammation through the production of inflammatory cytokines (Sarsour et al., 2009; Sánchez-Valle et al., 2012). In this regard, inflammatory cytokines such as TNF-α were reported to be extensively involved in the process of liver fibrogenesis (Handa and Kowdley, 2013). Inflammatory mediators could contribute to the eruption of oxidative stress and ROS generation by hindering cellular antioxidative mechanisms (Li et al., 2016). Accordingly, inflammation and oxidative stress interact together forming a vicious cycle creating an appropriate environment for liver fibrogenesis. In addition, ROS modulate signaling downstream of TGF-β1 as well as activation of the multicomponent NADPH oxidase complex (De Bleser et al., 1999; Seki et al., 2001).

Our findings did not confirm that plain ITRCZ dispersion mitigated oxidative stress because it failed to increase the levels of GSH and Catalase and demonstrated only mild improvement in SOD. However, ITRCZ-Cht NPs achieved the restoration of redox homeostasis. These latter effects can be attributed to the chitosan’s ability to act as a scavenger of free radicals due its amino and hydroxyl groups (Aranaz et al., 2021). In addition, the improved health status of liver tissue and the decreased inflammatory response contributed to the improvements observed in oxidative stress. However, based on our data we can not conclude that itraconazole is an antioxidant.

On the molecular level, findings of the current study highlighted the deregulation of the Hedgehog pathway in hepatic tissues of TAcA-intoxicated rats confirming their implication in hepatic fibrogenesis. Meanwhile, such deregulations were significantly modulated by ITRCZ-Cht NPs administration suggesting the capacity of ITRCZ-Cht NPs to lessen TAcA noxious effects by curtailing aberrant activation of Hedgehog. Our data suggest the capacity of ITRCZ-Cht NPs to modulate the potential crosstalk between Hedgehog and other signaling molecules (TGF-β, TIMP-1, PDGF-BB) in liver fibrosis, however, further experimental work is required to confirm this hypothesis.

Injury-related hyperactivation of the Hedgehog pathway was reported to play a pivotal role in promoting hepatic inflammation and fibrosis (Pibiri and Simbula, 2022). Specifically, Hedgehog pathway deregulation was reported to trigger principal facets of fibrogenic liver repair including quiescent HSCs trans-differentiation into myofibroblasts, modulating repair-related inflammatory responses as well as recruitment of inflammatory cells (Choi et al., 2011). Aberrant injury-induced activation of the Hedgehog pathway subsequent to chronic liver injury is mediated by abnormal excessive secretion of the pathway ligand by necrotic hepatic cells alongside decreased expression of Hhip, the antagonist of Hedgehog signaling (Chuang and McMahon, 1999). Once ligands bind to the pathway transmembrane receptor, this relieves receptor-mediated inhibition of SMO allowing its aggregation followed by nuclear localization of the transcription factors Gli1 and Gli2 with subsequent modulation of target genes expressions. Remarkably, each of the GLIs exerts a distinctive function where GLI2 is the main pathway activator, and GLI1 functions as a signal amplifier (Robbins et al., 2012).

Our results align well with previous reports that highlighted the attenuation of liver fibrosis through abrogation of the Hedgehog pathway (Jiayuan et al., 2020; Zhang et al., 2020). Moreover, Michelotti et al. (2013) reported that SMO deletion from α-SMA^+^ myofibroblasts caused inhibition of liver fibrosis. In addition, genetic deletion of GLI1 was associated with significant resolution of organ fibrosis (Kramann et al., 2015). Furthermore, the Hedgehog pathway represents a fundamental fibrogenic signaling pathway and a key component of a complex signaling network that includes other signaling pathways such as TGF-β signaling. Within this network, the Hedgehog pathway was reported to orchestrate other cascades regulating liver regeneration and fibrogenesis (He and Dai, 2015). Notably, GLI could regulate the transcription of the TGF-β signaling cascade target gene (Katoh and Katoh, 2008).

Remarkably, current findings are in agreement with an earlier *in-vitro* study that demonstrated the capacity of ITRCZ to suppress rat HSCs differentiation through inhibition of TGF-β, highlighting the potential for ITRCZ to be used in the treatment of fibrosis (Bollong et al., 2017b). Notably, TGF-β is a profibrogenic cytokine functioning in an autocrine loop manner and its role in promoting liver fibrosis is well established. Primarily, TGF-β deregulation contributes to the development of liver fibrosis through HSC activation, epithelial-to-mesenchymal transition, along excessive ECM deposition (Dewidar et al., 2019). Remarkably, aberrant TGF-β activation, after chronic liver damage, enhances the expression of fibrosis-linked proteins including α-SMA (Hinz et al., 2012; Zeng et al., 2015).

In conclusion, our findings highlighted that Hedgehog signaling is aberrantly activated in liver fibrosis development. Additionally, our work displayed, for the first time, the potential therapeutic benefit of ITRCZ in mitigating liver fibrosis that was likely ascribed to the reported modulation of the Hedgehog pathway. Moreover, this study is the first to investigate the potential antifibrotic effect of ITRCZ-Cht NPs as a new strategy to overcome the limitations of using plain ITRCZ dispersion. These observations lay the first stone for the development of a novel antifibrotic approach in the treatment of liver fibrosis using ITRCZ-Cht NPs.

## Data Availability

The original contributions presented in the study are included in the article/supplementary material, further inquiries can be directed to the corresponding author.

## Abbreviations

ALP, alkaline phosphatase; ALT, alanine aminotransferase; AST, aspartate aminotransferase; CAT, catalase; DSC, differential scanning calorimetry; ECM, extracellular matrix; EE%, entrapment efficiency; GLI, glioma-associated oncogene homolog; GSH, reduced glutathione; HSCs, hepatic stellate cells; ITRCZ, Itraconazole; ITRCZ-Cht NPs, chitosan nanoparticles loaded with itraconazole; LD%, loading efficiency; LDH, lactate dehydrogenase; MDA, malondialdehyde; NFκB, nuclear factor kappa B; PDGF-BB, platelet derived growth factor BB; PS, particle size; RT, room temperature; SMO, Smoothened; SOD, super oxide dismutase; TAcA, thioacetamide; TEM, transmission electron microscope; TGF-β, transforming growth factor beta; TIMP-1, tissue inhibitor of metalloproteinase-1; ZP, zeta potential; α-SMA, α-smooth muscle actin; γGT, gamma glutamyl transferase.
